# Roadmap on magnetic nanoparticles in nanomedicine

**DOI:** 10.1088/1361-6528/ad8626

**Published:** 2024-11-05

**Authors:** Kai Wu, Jian-Ping Wang, Niranjan A Natekar, Stefano Ciannella, Cristina González-Fernández, Jenifer Gomez-Pastora, Yuping Bao, Jinming Liu, Shuang Liang, Xian Wu, Linh Nguyen T Tran, Karla Mercedes Paz González, Hyeon Choe, Jacob Strayer, Poornima Ramesh Iyer, Jeffrey Chalmers, Vinit Kumar Chugh, Bahareh Rezaei, Shahriar Mostufa, Zhi Wei Tay, Chinmoy Saayujya, Quincy Huynh, Jacob Bryan, Renesmee Kuo, Elaine Yu, Prashant Chandrasekharan, Benjamin Fellows, Steven Conolly, Ravi L Hadimani, Ahmed A El-Gendy, Renata Saha, Thomas J Broomhall, Abigail L Wright, Michael Rotherham, Alicia J El Haj, Zhiyi Wang, Jiarong Liang, Ana Abad-Díaz-de-Cerio, Lucía Gandarias, Alicia G Gubieda, Ana García-Prieto, Mª Luisa Fdez-Gubieda

**Affiliations:** 1 Department of Electrical and Computer Engineering, Texas Tech University, Lubbock, TX, United States of America; 2 Department of Electrical and Computer Engineering, University of Minnesota, Minneapolis, MN, United States of America; 3 Western Digital Corporation, San Jose, CA, United States of America; 4 Department of Chemical Engineering, Texas Tech University, Lubbock, TX, United States of America; 5 Department of Chemical and Biomolecular Engineering, University of Cantabria, Santander, Spain; 6 Department of Chemical and Biological Engineering, The University of Alabama, Tuscaloosa, AL, United States of America; 7 Department of Chemical Engineering and Materials Science, University of Minnesota, Minneapolis, MN, United States of America; 8 William G Lowrie Department of Chemical and Biomolecular Engineering, The Ohio State University, Columbus, OH, United States of America; 9 National Institute of Advanced Industrial Science and Technology (AIST), Health and Medical Research Institute, Tsukuba, Ibaraki 305-8564, Japan; 10 Department of Electrical Engineering and Computer Sciences, University of California Berkeley, Berkeley, CA, United States of America; 11 Department of Bioengineering, University of California Berkeley, Berkeley, CA, United States of America; 12 Magnetic Insight, Alameda, CA, United States of America; 13 Department of Mechanical and Nuclear Engineering, Virginia Commonwealth University, Richmond, VA, United States of America; 14 Department of Biomedical Engineering, Virginia Commonwealth University, Richmond, VA, United States of America; 15 Department of Psychiatry, Harvard Medical School, Harvard University, Boston, MA, United States of America; 16 Department of Physics, University of Texas at El Paso, El Paso, TX, United States of America; 17 Healthcare Technologies Institute, School of Chemical Engineering, University of Birmingham, Edgbaston, Birmingham, United Kingdom; 18 National Institute for Health and Care Research (NIHR) Birmingham Biomedical Research Centre, Institute of Translational Medicine, Birmingham, United Kingdom; 19 Spin-X Institute, School of Chemistry and Chemical Engineering, State Key Laboratory of Luminescent Materials and Devices, South China University of Technology, Guangzhou, Guangdong Province, People’s Republic of China; 20 Dpto. Inmunología, Microbiología y Parasitología, Universidad del País Vasco–UPV/EHU, Leioa, Spain; 21 Bioscience and Biotechnology Institute of Aix-Marseille (BIAM), Aix-Marseille Université, CNRS, CEA—UMR 7265, Saint-Paul-lez-Durance, France; 22 Dpto. Electricidad y Electrónica, Universidad del País Vasco—UPV/EHU, Leioa, Spain; 23 Dpto. Física Aplicada, Universidad del País Vasco–UPV/EHU, Bilbao, Spain

**Keywords:** magnetic nanoparticle, biomedical application, magnetic imaging, magnetic biosensing, hyperthermia, tissue engineering, drug delivery

## Abstract

Magnetic nanoparticles (MNPs) represent a class of small particles typically with diameters ranging from 1 to 100 nanometers. These nanoparticles are composed of magnetic materials such as iron, cobalt, nickel, or their alloys. The nanoscale size of MNPs gives them unique physicochemical (physical and chemical) properties not found in their bulk counterparts. Their versatile nature and unique magnetic behavior make them valuable in a wide range of scientific, medical, and technological fields. Over the past decade, there has been a significant surge in MNP-based applications spanning biomedical uses, environmental remediation, data storage, energy storage, and catalysis. Given their magnetic nature and small size, MNPs can be manipulated and guided using external magnetic fields. This characteristic is harnessed in biomedical applications, where these nanoparticles can be directed to specific targets in the body for imaging, drug delivery, or hyperthermia treatment. Herein, this roadmap offers an overview of the current status, challenges, and advancements in various facets of MNPs. It covers magnetic properties, synthesis, functionalization, characterization, and biomedical applications such as sample enrichment, bioassays, imaging, hyperthermia, neuromodulation, tissue engineering, and drug/gene delivery. However, as MNPs are increasingly explored for *in vivo* applications, concerns have emerged regarding their cytotoxicity, cellular uptake, and degradation, prompting attention from both researchers and clinicians. This roadmap aims to provide a comprehensive perspective on the evolving landscape of MNP research.

## Introduction

In recent years, the study of nanomaterials has gone beyond traditional scientific boundaries, opening a new era of possibilities across various disciplines. Among these, magnetic nanoparticles (MNPs) have emerged as a popular class of nanomaterials, exhibiting unique physicochemical properties due to their nanoscale dimensions and magnetic compositions. MNPs are composed of magnetic cores made from materials such as iron, cobalt, nickel, or their alloys, along with one or more organic/inorganic shells. They are typically exhibiting diameters within the range of 1–100 nanometers.

In the last decade, MNPs have found applications not only in the biomedical domain but also in environmental remediation, data storage, energy storage, and catalysis. This roadmap serves as a comprehensive guide, navigating through the current landscape of MNPs in nanomedicine research. Each section in this roadmap unfolds a distinct facet of MNPs, from understanding their magnetic properties to exploring innovative biomedical applications such as sample enrichment, bioassays, medical imaging, hyperthermia, neuromodulation, tissue engineering, and drug/gene delivery. Furthermore, the roadmap addresses crucial aspects like cellular uptake and degradation, providing a holistic perspective on the evolving landscape of MNP research in the dynamic field of nanomedicine.

Section 1 lays the groundwork for this roadmap by introducing fundamental knowledge about MNPs. Key parameters such as saturation magnetization and magnetic anisotropy are introduced. Compared to their bulk counterparts, MNPs usually show lower saturation magnetization and higher anisotropy due to the spin canting effect. In many applications, MNPs experience rapidly changing magnetic fields, particularly alternating magnetic fields. This dynamic environment poses challenges in predicting and modeling the responses of an ensemble of MNPs, considering coupled Néel and Brownian relaxations, along with dipole-dipole interactions. Various mathematical models have been developed to explain the dynamic magnetizations of MNPs, including the Stoner Wohlfarth (SW) model, static Langevin model, Debye model, Landau–Lifshitz–Gilbert (LLG) model, stochastic Langevin model, and Fokker-Planck model. Each model comes with its own set of advantages and limitations, making them suitable for different external field conditions.

The synthesis and functionalization of MNPs have been the focus of extensive research to tailor their properties for specific applications. Thus, in sections 2–4, we have covered the synthesis, surface functionalization, and characterization methods for MNPs. The continual effort to improve the chemical and magnetic properties of MNPs is driving advancements in their medical applications. Synthesis methods are crucial in shaping key tunable aspects like size, morphology, surface chemistry, and magnetic properties. Section 2 discussed various synthesis methods, encompassing physical, chemical, and biological approaches. Surface functionalization plays a vital role in shaping the physical and chemical properties of MNPs and determining their potential applications. This process not only ensures the colloidal stability and biocompatibility of MNPs but also influences their interactions with biological systems, impacting behaviors such as cellular uptake for cell-based therapy. Over the last decade, significant strides have been made, presenting opportunities for improved biocompatibility, minimal opsonization, precise targeting, and enhanced single-cell sensitivity in imaging and diagnosis. In section 3, the critical role of MNP surface functionalization in biomedical applications is emphasized, along with an acknowledgment of current limitations. Specifically, it highlights the absence of standardized characterization techniques, qualification strategies, and the need for greater reproducibility across studies in the current state of MNPs’ surface functionalization. Finally, the characterization of MNPs is essential to guarantee that their inherent properties align with the diverse demands of biomedical applications. Section 4 provides an overview of prevalent characterization techniques employed to gather information on MNPs, including their size and morphology, structure and composition, colloidal stability, magnetic properties, and more. However, it is crucial to acknowledge that specific characterization techniques present challenges that demand meticulous attention. In addition to conventional methods, this section explores several advanced techniques designed to tackle current challenges in MNP characterization.

Sections 5 and 6 cover the MNP-based magnetic enrichment and bioassays. Magnetic separation is a critical technique in biomedical fields, facilitating sample enrichment for diagnostics, therapeutics, and cellular biology research. This method efficiently isolates and purifies target substances based on their magnetic properties (either magnetically labeled by MNPs or label-free). It is especially valuable for recovering and analyzing target bio-entities present at ultra-low concentrations, particularly in the context of early disease diagnosis. However, the complexity of the magnetic separation process involves various factors influencing material behavior under an external magnetic field. In section 5, the authors address key challenges for both types of magnetic separation filters to enhance performance and throughput, along with recent advances in the field. Considering the nonmagnetic nature of most biological samples, employing MNPs as labels for detecting biological target analytes offers an inherent advantage, resulting in minimal background noise and, consequently, enhanced detection limits compared to analogous chemical or optical labels. In section 6, various magnetic biosensors are examined, and diverse bioassay mechanisms are detailed. Challenges associated with the transformation of magnetic biosensors into point-of-care applications are discussed. With the increasing demand for multiplexing bioassays that are faster, more sensitive, and cost-effective, there is a need for fully automatic assays that require minimal effort from users.

Medical imaging is a crucial component of contemporary healthcare, offering valuable insights into the structure and function of the human body in a minimally invasive manner. Two prominent medical imaging techniques currently leveraging MNPs are magnetic resonance imaging (MRI) and magnetic particle imaging (MPI). MNPs are commonly used as contrast agents in MRI to improve visibility, and their application as T1, T2, or dual T1/T2 contrast agents was discussed in section 7. While gadolinium-based contrast agents are widely used, they pose risks such as nephrogenic systemic fibrosis and brain deposition. Researchers are exploring manganese as a T1-weighted alternative, but its toxicity raises concerns. Ongoing research focuses on iron-oxide-based MNPs as biocompatible contrast agents and drug carriers, despite susceptibility artifacts in T2-weighted images, emphasizing the need for colloidal stability in developing new dual-contrast MR agents.

Section 8 discussed MPI, which is an emerging imaging technique distinct from MRI in hardware and physics, offering excellent safety, contrast, sensitivity, and robustness. Despite being the only non-radioactive deep tissue ‘reporter/tracer’ imaging method, MPI faces challenges such as superparamagnetism limitations on the MNP tracers’ sizes and lower spatial resolution compared to computed tomography (CT), MRI, and ultrasound. Ongoing efforts involve utilizing tailored superferromagnetic nanoparticle tracers with steeper magnetization curves and smaller saturation fields, leading to significant improvements in both MPI resolution and signal-to-noise ratio. Strategies to extend the circulation half-life of MNP tracers and employ active approaches like diapedesis by tumor-associated immune cells are proposed to enhance the targeted delivery of MNPs, improving the specificity of MPI.

Sections 9–12 are a collection of therapeutic applications of MNPs. Magnetic hyperthermia is a popular therapy tool for cancer treatment, yet its effectiveness is hindered by challenges in achieving uniform heat distribution during tumor-specific thermal treatment. Self-regulating magnetic hyperthermia emerges as a promising solution to ensure consistent heating throughout the targeted tissue. In section 9, the current state of self-regulating magnetic hyperthermia was discussed, relying on the premise that MNPs possess a magnetic transition temperature (*T*_C_) around the intended treatment temperature, ensuring magnetic heating is confined to that specific temperature range. Despite its potential, the synthesis of well-dispersed self-regulating magnetic hyperthermia nanoparticles presents a considerable challenge, mainly due to the limited availability of magnetic materials with transition temperatures close to the body temperature, which is vital for the efficacy of hyperthermia treatment.

When subjected to an alternating magnetic field (AMF), MNPs generate heat, mechanical force, and/or electric fields at the nanoscale. The ion channels in neurons are responsive to these types of external stimuli mediated by MNPs, as detailed in section 10. Various existing MNP-mediated neuromodulation techniques, including magnetothermal, magnetoelectric, chemomagnetic, and magnetogenetic methods, facilitate the opening of ion channels during stimulation, allowing ions to enter neuronal cells and induce excitation or inhibition. MNP-mediated neuromodulation offers non-invasive access to deep brain regions with high specificity, providing insights into neural circuits, motor behaviors, and potential treatments for neuropsychiatric disorders. Despite progress, MNP-mediated neuromodulation is at an early stage and requires further refinement to advance applications in neuroscience and move toward clinical trials, as emphasized by the authors.

Tissue engineering (TE) replaces the diseased or damaged tissue that cannot be treated by conventional therapies, with MNPs emerging as a crucial component of the TE toolkit. In section 11, the authors discussed MNPs’ role in steering the growth and alignment of cells and tissues, as well as their ability to initiate mechanotransduction across various cell types by MNP-induced movement. Simultaneously, with magnetic imaging techniques like MRI and MPI, MNPs offer a non-invasive means of imaging and monitoring the integration of engineered tissue constructs. Despite these advancements, challenges in utilizing MNPs for regenerative TE persist, including concerns about biosafety, the spatial distribution of magnetic fields, and the precise repair and regeneration of functional tissues containing multiple cell types arranged in the correct sequence.

MNPs’ intrinsic magnetic properties, which make them responsive to external magnetic fields, are crucial for targeted drug and gene delivery, especially in deep body locations like the brain. Section 12 discusses the development of MNP assemblies, polymers, liposomes, and silane coupling agents as drug carriers, demonstrating the potential for controlled and targeted release, especially in cancer therapy. The creation of magnetically engineered drug delivery systems (MEDDS) that integrate MNPs with other materials is essential to facilitate selective and conditional drug release, ensuring targeted treatment with minimal harm to healthy cells.

As the interest in MNPs for *in vivo* applications intensifies, it is important to understand the cellular uptake mechanisms of MNPs to enhance delivery efficacy to target cells and prevent clearance by the immune system. Section 13 discussed diverse endocytic pathways through which MNPs enter cells. The physicochemical properties of MNPs influence both their cellular uptake and degradation rate. For instance, smaller MNPs degrade faster due to their higher surface area-to-volume ratio. Additionally, multicore MNPs exhibit a faster degradation rate compared to core-shell multicore MNPs. Upon entering cells, understanding the effect of MNP degradation on their therapeutic and diagnostic potential becomes pivotal. Researchers strive to precisely locate and quantify MNPs, identifying their degradation products to gain deeper insights into the degradation process and its implications in cancer treatment. While the classical approach to studying MNP endocytosis pathways has certain limitations, novel microscopy techniques provide several options for tracking the internalization and degradation of MNPs at a cellular level.

Kai Wu and Jian-Ping Wang

Editors of the Roadmap on Magnetic Nanoparticles in Nanomedicine

## 1. Magnetic properties and dynamic magnetizations of MNPs

### Niranjan A Natekar^1^ and Kai Wu^2^

^1^ Western Digital Corporation, San Jose, CA, United States of America

^2^ Department of Electrical and Computer Engineering, Texas Tech University, Lubbock, TX, United States of America

### Status

**Figure 1.**
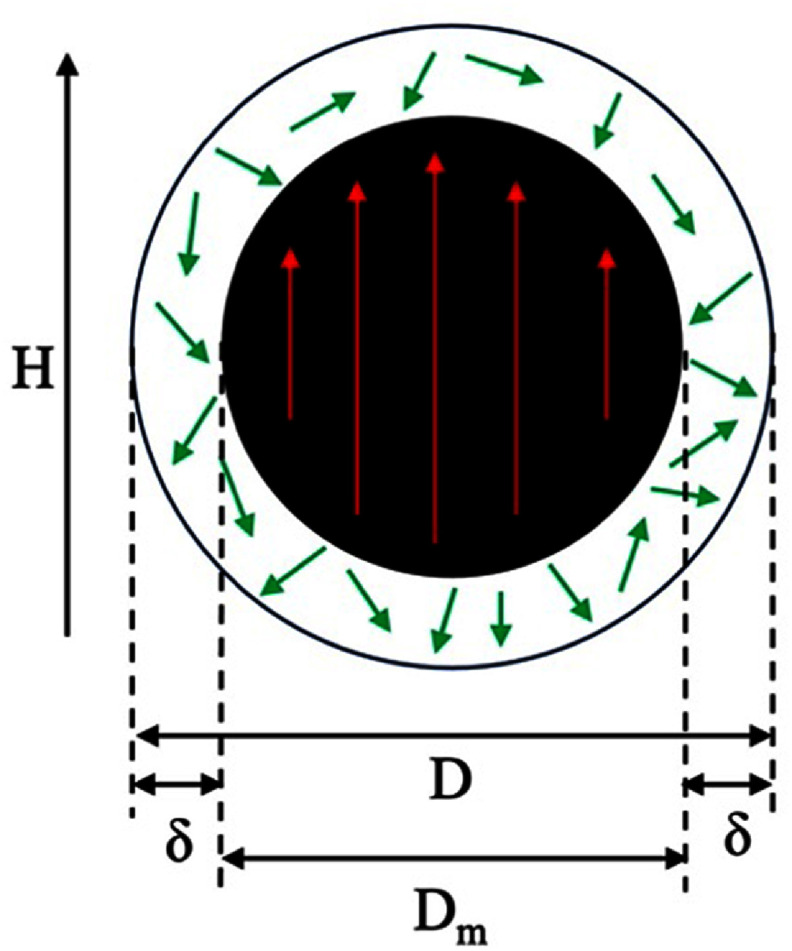
One MNP with a core diameter of *D*. The spin-ordered part of the core has a diameter of *D*_m_, with a surface spin disorder in a spin-canted layer of thickness $\delta $. Original figure prepared by the authors.

Magnetic nanoparticles (MNPs) refer to particles with a magnetic core size of up to 100 nm. MNPs find applications in several areas including information and energy storage, magnetic imaging and biosensors, medical applications, and drug and gene delivery systems. The magnetic properties of MNPs differ from their bulk counterparts. One characteristic of MNPs is their small size and high surface-to-volume ratio. The surface canting effect describes the zeroing of the magnetization due to the canted alignment of spins. In figure 1, atoms inside the spin-ordered portion of the core (with diameter *D*_m_) maintain their magnetizations based on the external field. As the size of the MNP decreases, the saturation magnetization (*M*_s_) of the MNP decreases compared to the bulk value. This reduction is proportional to the difference in core and magnetic diameter of the MNP. The spin canting effect can occur due to (i) the breaking of the crystallographic symmetry at the MNP surface (2) the change in lattice structure at the MNP surface, and (3) the reduced coordination and exchange bonds of atoms at the MNP surface. The effective crystalline anisotropy (*K*_eff_) is also affected by the reduction in the MNP size. Equation (1) shows how *K*_eff_ increases as the MNP core size (D) reduces [1]:

\begin{align*}{K_{{\text{eff}}}} = {K_{\text{V}}} + \frac{6}{D}{K_{\text{S}}} \end{align*}

The volume component of crystalline anisotropy, *K*_v_, is the energy difference between the default magnetization state and the state in the presence of an external field. The presence of surface anisotropy *K*_s_ is valid only when spin-orbit coupling (SOC) and surface anisotropy are strong. Similarly, the cancelation of anisotropy perpendicular to the MNP surface, valid for symmetrical MNP shapes like spheres and cubes makes equation (1) invalid for these shapes. For anisotropic MNPs such as elongated shapes, plate-like shapes, etc, their effective anisotropy is more complicated, readers can refer to this work in [2].

The previous discussions are highly qualitative and primarily focused on the effect of spin canting on the magnetic properties of MNPs in an idealized scenario. Surface coatings of organic or inorganic materials, chemical binding, and other factors can alter this spin canting layer [3, 4]. Additionally, defects in the crystalline structure, annealing history, temperature, and inter-particle interactions also affect the magnetic properties of MNPs. For nanoscale materials, atomic-level to inter-particle level interactions, along with the material’s history, make it difficult to model their magnetic properties accurately. As a result, the reported magnetic properties of MNPs vary widely. For instance, reported magnetic anisotropy values for iron oxide MNPs range from 20 to 100 kJ m^−3^ [5], and saturation magnetizations vary from 0 to the bulk iron oxide’s value, which is 80–100 Am^2^ kg^−1^ [6].

To date, most applications of MNPs are governed by their unique dynamic magnetic properties. Various models have been developed to describe the magnetic behaviors of MNPs, including the earliest Stoner–Wohlfarth (SW) model, Langevin function, Debye model, and Landau–Lifshitz–Gilbert (LLG) equation. These models often ignore one or more energy terms or isolate only one of the Néel and Brownian relaxations, which limits their applicability to ideal scenarios and makes them less suitable for real-life applications. In the past 30 years, new models such as the stochastic Langevin [7] and the Fokker–Planck [8], which consider the coupled Néel and Brownian relaxations, have been proposed and shown to be accurate for studying the dynamic magnetizations of MNPs.

### Current and future challenges

**Figure 2.**
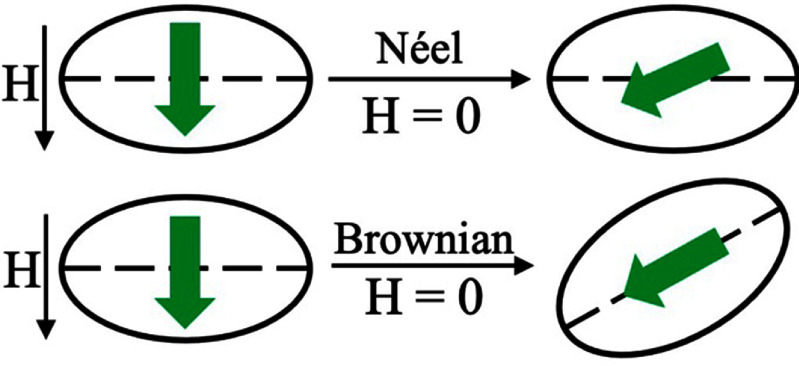
Schematic drawing representing the zero-field Brownian and Néel relaxations. The dotted line represents the easy axis of the MNP. Original figure prepared by the authors.

As mentioned before, the early models such as SW, Langevin, Debye, and LLG equations have their individual shortness. In this roadmap, these models are not elaborated. We will focus on the recent 20–30 yr’ attempts to model the dynamics magnetizations of MNPs. This part starts with the Néel and Brownian relaxation models at equilibrium (see figure 2). These equilibrium relaxations are only valid for DC and slowly varying AC fields and assume both relaxation mechanisms are decoupled.

Nonequilibrium models are proposed to overcome the limitations associated with the magnetization dynamics of MNPs under rapidly varying AC fields. Which applies to most MNP-based applications nowadays such as hyperthermia, MPI, etc. Based on the Debye model, the empirical Brownian and Néel relaxation models are derived by Yoshida *et al* [9] and Dieckhoff *et al* [10] from experimental data fitting. Like the equilibrium models, these empirical models assume the Brownian and Néel relaxations to be decoupled and only one relaxation mechanism dominates at a time.

Later, the Fokker–Planck equation, initially used by Brown [11] to describe the time evolution of the probability density function for the orientation angles of an ensemble of MNPs, has been modified by various groups to study the decoupled Brownian [12, 13] and decoupled Néel [14] relaxations using effective field approximations. Despite these modifications, whether through empirical methods or effective field approximations, the Brownian and Néel relaxations are still treated as independent and decoupled processes. This treatment invalidates these models and their variants for accurately computing the behavior of MNPs in real biological environments.

### Advances in science and technology to meet challenges

The models introduced in previous sections define the magnetization dynamics of MNPs with decoupled relaxation mechanisms under restrictions of field profiles. The solution to these limitations is to consider coupled Brownian and Néel relaxations. To counter this limitation, Brownian relaxation is added to the stochastic Langevin and Fokker-Planck equations by considering the orientation dynamics of the easy axis.

For both these formulations, the effective field equation in the model can be solved numerically, but the formulation assumes that the applied field and anisotropy axis are coaxial along the *z*-axis. In the stochastic Langevin equation, the LLG equation includes the dynamics of the magnetization unit vector direction, $\hat m$, namely, the Néel relaxation. The total energy of the system governs the Brownian relaxation dynamics of the MNP’s with direction specified by $\hat n$ (taking the easy axis as reference). The coupled time evolution of both relaxations is expressed as [7, 15, 16]:

\begin{align*} \frac{{{\textrm{d}}\widehat {\boldsymbol{m}}}}{{{\textrm{d}}t}} &amp;= - \gamma \widehat {\boldsymbol{m}} \times {\mu _0}{{\boldsymbol{H}}_{{\boldsymbol{\text{eff}}}}} + \alpha \widehat {\boldsymbol{m}} \times \frac{{{\textrm{d}}\widehat {\boldsymbol{m}}}}{{{\textrm{d}}t}}{\textrm{ }}\nonumber\\ \frac{{{\textrm{d}}\widehat {\boldsymbol{n}}}}{{{\textrm{d}}t}}&amp; = \frac{{\boldsymbol{\Theta }}}{{6\eta {V_h}}} \times \widehat {\boldsymbol{n}}\end{align*}

where $\gamma $ is the gyromagnetic ratio, ${{\boldsymbol{H}}_{{\boldsymbol{\text{eff}}}}}$ effective field (a combination of the applied field and other interacting fields), $\alpha $ is the LLG damping parameter, ${\boldsymbol{\Theta }}$ is the torque acting on the particle, ${V_h}\,$ is the hydrodynamic volume of the particle and $\eta $ is the viscosity of the fluid.

Considering the thermal fluctuations, the total energy and torque can be expressed as:

\begin{equation*}{{\boldsymbol{H}}_{{\boldsymbol{\mathbf{eff}}}}} = - \frac{1}{{{\mu _0}{M_S}{V_C}}}\frac{{\partial E}}{{\partial \widehat {\boldsymbol{m}}}} = {{\boldsymbol{H}}_{\boldsymbol{a}}} + \frac{{2{K_u}{V_C}}}{{{\mu _0}{M_S}{V_C}}}\left( {\widehat {\boldsymbol{m}} \cdot \widehat {\boldsymbol{n}}} \right)\widehat {\boldsymbol{n}}\end{equation*}

\begin{equation*}{\boldsymbol{\Theta }} = \frac{{\partial E}}{{\partial \widehat {\boldsymbol{n}}}} \times \widehat {\boldsymbol{n}} = 2{K_u}{V_{\text{C}}}\left( {\widehat {\boldsymbol{m}} \cdot \widehat {\boldsymbol{n}}} \right)\left( {\widehat {\boldsymbol{m}} \times \widehat {\boldsymbol{n}}} \right),\end{equation*}

where ${V_{\text{C}}}$ is the magnetic core volume of the particle, ${M_{\text{S}}}$ and ${K_u}$ refer to the magnetic properties (saturation magnetization and anisotropy, respectively), and ${{\boldsymbol{H}}_{\boldsymbol{a}}}$ is the applied field.

The stochastic simulations based on equations (2)–(4) can be numerically solved to model the coupled Brownian and Néel relaxations of MNP ensembles.

In 2017, Weizenecker derived the Fokker–Planck equation for the coupled Brownian and Néel relaxations, based on the coupled Langevin equations (equations (5) and (6)). The uniaxial anisotropy and magnetization direction are defined in a spherical coordinate system as shown below, with four degrees of freedom. The coupled Fokker-Planck equation is expressed below [8]:

\begin{equation*}\widehat {\boldsymbol{n}} = {N_x}\widehat {\boldsymbol{x}} + {N_y}\widehat {\boldsymbol{y}} + {N_z}\widehat {\boldsymbol{z}} = {N_\rho }{\widehat {\boldsymbol{e}}_{\boldsymbol{\rho }}} + {N_\theta }{\widehat {\boldsymbol{e}}_{\boldsymbol{\theta }}} + {N_\phi }{\widehat {\boldsymbol{e}}_\phi }\end{equation*}

\begin{equation*}\widehat {\boldsymbol{m}} = {U_x}\widehat {\boldsymbol{x}} + {U_y}\widehat {\boldsymbol{y}} + {U_z}\widehat {\boldsymbol{z}} = {U_r}{\widehat {\boldsymbol{e}}_{\boldsymbol{r}}} + {U_\vartheta }{\widehat {\boldsymbol{e}}_{\boldsymbol{\vartheta}} } + {U_\varphi }{\widehat {\boldsymbol{e}}_{\boldsymbol{\varphi }}},\end{equation*}

where $\widehat {\boldsymbol{n}}$ and $\widehat {\boldsymbol{m}}$ are the unit vectors in the anisotropy and magnetic moment directions, respectively. Both these vectors are represented in the spherical coordinate system based on the angles they make with the coordinate axes. *N* and *U* represent the magnitudes in different directions for the anisotropy and moment, respectively.

A change in the particle easy axis direction or a change in the moment will lead to a change in the torque and the resulting angular momentum. The equations of motion for both the direction of the particle and the moment can be written by using the definition of generalized torques that will add friction terms to the Euler–Lagrange function. This results in the system of equations represented by equations (7) and (8) below:

\begin{equation*}\frac{{{\text{d}}\vec n}}{{{\text{d}}t}} = { }\frac{{{K_u}{V_{\text{c}}}}}{{3\eta {V_h}}}\left( {\vec n \cdot \vec m} \right)\left[ {\left( {\vec n \times \vec m} \right) \times \vec n} \right] + { }\frac{1}{{\sqrt {{\tau _{\text{B}}}} }}\vec Y\left( t \right) \times \vec n\end{equation*}

\begin{align*}\frac{{{\text{d}}\vec m}}{{{\text{d}}t}}&amp; = \frac{1}{{2{\tau _{\text{N}}}}}\frac{{{M_{\text{s}}}{V_{\text{c}}}}}{{\alpha {k_{\text{B}}}T}}\left\{ \left[ {\vec m \times \left( {\vec H + \frac{{2{K_u}}}{{{M_{\text{s}}}}}\left( {\left( {\vec m \cdot \vec n} \right)\vec n} \right)} \right)} \right]\right. \nonumber\\ &amp; \left. \quad + \left[ {\vec m \times \left( {\vec H + \frac{{2{K_u}}}{{{M_{\text{s}}}}}\left( {\left( {\vec m \cdot \vec n} \right)\vec n} \right)} \right)} \right] \times \vec m \right\}\nonumber\\ &amp;\quad + \frac{1}{{\sqrt {\left( {1 + {\alpha ^2}} \right){\tau _{\text{N}}}} }}\left[ {\vec m \times \vec X + \left( {\vec m \times \vec X} \right) \times \vec m} \right].\end{align*}

In equations (7) and (8), *η* is the viscosity of the fluid, $\alpha $ is the LLG damping parameter for the magnetic moment, $\vec H$ is the vector field, *T* is the absolute temperature and *k*_B_ is the Boltzmann constant. ${\tau _{\text{B}}}$ in equation (7) and ${\tau _{\text{N}}}$ in equation (8) are time constants and are defined below in equation (9):

\begin{equation*}{\tau _{\text{B}}} = {\mkern 1mu} \frac{{3\eta {V_h}}}{{{k_{\text{B}}}T}},{\mkern 1mu} {\mkern 1mu} {\tau _{\text{N}}} = {\mkern 1mu} \frac{{{M_{\text{s}}}{V_{\text{c}}}}}{{{k_{\text{B}}}T\gamma }}\frac{{1 + {\alpha ^2}}}{{2\alpha }}{\mkern 1mu} .\end{equation*}

The stochastic torques *X* and *Y* related to the Brown rotation and Néel rotation, respectively, and are related as follows:

\begin{align*}\begin{aligned} \left\langle {{X_i}\left( t \right){X_j}\left( {t{^{^{\prime}}}} \right)} \right\rangle \,&amp; = \,{\delta _{ij}}\delta \left( {t - t{^{^{\prime}}}} \right) \\ \left\langle {{Y_i}\left( t \right){Y_j}\left( {t{^{^{\prime}}}} \right)} \right\rangle \,&amp; = \,{\delta _{ij}}\delta \left( {t - t^{\prime}} \right) \\ \left\langle {{X_i}\left( t \right)} \right\rangle \, = \,\left\langle {{Y_i}\left( t \right)} \right\rangle \,&amp; = \,\left\langle {{X_i}\left( t \right){Y_j}\left( {t{^{^{\prime}}}} \right)} \right\rangle \, = 0 \end{aligned} .\end{align*}

Equations (7) and (8) together possesses four degrees of freedom. Since $\vec n$ and $\vec m$ are independent, they are expressed in two different spherical coordinate systems. The angles $\vartheta $ and $\varphi $ define the direction of $\vec n$ whereas $\theta $ and $\phi $ define the direction of $\vec m$. The following relations stand true for the derivatives of $\vec n$ and $\vec m$:

\begin{equation*}\frac{{{\text{d}}\vec n}}{{{\text{d}}t}} = \frac{{{\text{d}}\overrightarrow {{m_r}} }}{{{\text{d}}t}} = { }\frac{{{\text{d}}\vartheta }}{{{\text{d}}t}}{ }\overrightarrow {{m_\vartheta }} + \sin \left( \vartheta \right)\frac{{{\text{d}}\varphi }}{{{\text{d}}t}}\overrightarrow {{m_\varphi }} \end{equation*}

\begin{equation*}\frac{{{\text{d}}\vec m}}{{{\text{d}}t}} = \frac{{{\text{d}}\overrightarrow {{m_\rho }} }}{{{\text{d}}t}} = { }\frac{{{\text{d}}\theta }}{{{\text{d}}t}}{ }\overrightarrow {{m_\theta }} + \sin \left( \theta \right)\frac{{{\text{d}}\emptyset }}{{{\text{d}}t}}\overrightarrow {{m_\emptyset }} .\end{equation*}

The terms in equations (7) and (8) can be further calculated in terms of the spherical coordinate system within which the variables of the equation are represented. By comparing the pre-factors of the basis vectors from the calculations within equations (7) and (8) to the relations expressed above, we get four coupled equations as shown in equation (13):

\begin{align*} \frac{{{\textrm{d}}\vartheta }}{{{\textrm{d}}t}} &amp;= {\mkern 1mu} \frac{{{K_u}{V_{\textrm{c}}}}}{{{k_{\textrm{B}}}T}}\frac{1}{{{\tau _{\textrm{B}}}}}{U_r}{U_\vartheta } + \frac{1}{{\sqrt {{\tau _{\textrm{B}}}} }}{Y_\varphi } \nonumber \\ \sin \vartheta \frac{{{\textrm{d}}\varphi }}{{{\textrm{d}}t}}&amp; = {\mkern 1mu} \frac{{{K_u}{V_{\textrm{c}}}}}{{{k_{\textrm{B}}}T}}\frac{1}{{{\tau _{\textrm{B}}}}}{U_r}{U_\varphi } - \frac{1}{{\sqrt {{\tau _{\textrm{B}}}} }}{Y_\vartheta }\nonumber\\ \frac{{{\textrm{d}}\theta }}{{{\textrm{d}}t}} &amp;= {\mkern 1mu} \frac{1}{{2{\tau _{\textrm{N}}}}}\frac{{{M_{\textrm{s}}}{V_{\textrm{c}}}}}{{\alpha {k_{\textrm{B}}}T}}\left( {\alpha {H_\theta } - {H_\emptyset }} \right) + \frac{1}{{2{\tau _{\textrm{N}}}}}\frac{{2{K_u}{V_{\textrm{c}}}}}{{\alpha {k_{\textrm{B}}}T}}{{\textrm{N}}_\rho }\left( {\alpha {{\textrm{N}}_\theta } - {{\textrm{N}}_\emptyset }} \right)\;\nonumber\\ &amp; \quad + \;\frac{1}{{\sqrt {\left( {1 + {\alpha ^2}} \right){\tau _{\textrm{N}}}} }}\left( {\alpha {X_\theta } - {X_\emptyset }} \right)\nonumber\\ \sin \theta \frac{{{\textrm{d}}\emptyset }}{{{\textrm{d}}t}}&amp; = {\mkern 1mu} \frac{1}{{2{\tau _{\textrm{N}}}}}\frac{{{M_{\textrm{s}}}{V_{\textrm{c}}}}}{{\alpha {k_{\textrm{B}}}T}}\left( {{H_\theta } + \alpha {H_\emptyset }} \right) + \frac{1}{{2{\tau _{\textrm{N}}}}}\frac{{2{K_u}{V_{\textrm{c}}}}}{{\alpha {k_{\textrm{B}}}T}}{{\textrm{N}}_\rho }\left( {{{\textrm{N}}_\theta } + \alpha {{\textrm{N}}_\emptyset }} \right)\;\nonumber\\ &amp; \quad + \;\frac{1}{{\sqrt {\left( {1 + {\alpha ^2}} \right){\tau _{\textrm{N}}}} }}\left( {{X_\theta } + \alpha {X_\emptyset }} \right).\end{align*}

For a system of coupled stochastic differential equations with zero mean ($ \langle {h_i}\left( t \right) \rangle \, = 0$) and satisfying the Kronecker delta relation ($ \langle {h_i}\left( t \right){h_j}\left( {t{^{^{\prime}}}} \right) \rangle \, = {\delta _{ij}}\delta \left( {t - t^{\prime}} \right)\,$), it can be used to derive a stochastic partial differential equation. The drift and diffusion terms for this equation are calculated from the four coupled equations shown above (equation (13)). These drift and diffusion terms are quoted in [16].

Inserting the drift and diffusion terms in the stochastic partial differential equation allows us to get the Fokker Planck equations in the coupled form as shown below in equation (14):

\begin{align*} \frac{{\partial F}}{{\partial t}}\;&amp; = \; - \frac{1}{{2{\tau _{\textrm{B}}}}}\frac{1}{{\sin \vartheta }}\frac{\partial }{{\partial \vartheta }}\left[ {\frac{{2{K_u}{V_C}}}{{{k_{\textrm{B}}}T}}{U_r}{U_\vartheta }\sin \vartheta F - \sin \vartheta \frac{{\partial F}}{{\partial \vartheta }}} \right]\nonumber\\ &amp; \quad - \frac{1}{{2{\tau _{\textrm{B}}}}}\frac{1}{{\sin \vartheta }}\frac{\partial }{{\partial \varphi }}\left[ {\frac{{2{K_u}{V_C}}}{{{k_{\textrm{B}}}T}}{U_r}{U_\varphi }F} \right]\nonumber\\ &amp; \quad - \,\frac{1}{{2{\tau _{\textrm{N}}}}}\frac{1}{{\sin \theta }}\frac{\partial }{{\partial \theta }}\left[ \frac{{{\mu _0}{M_{\textrm{S}}}{V_{\textrm{C}}}}}{{\alpha {k_{\textrm{B}}}T}}\left( {\alpha {H_\theta } - {H_\phi }} \right)\sin \theta F\right. \nonumber\\ &amp; \quad \left. + \frac{{2{K_u}{V_{\textrm{C}}}}}{{\alpha {k_{\textrm{B}}}T}}{{\textrm{N}}_\rho }\left( {\alpha {{\textrm{N}}_\theta } - {{\textrm{N}}_\phi }} \right)\sin \theta F - \sin \theta \frac{{\partial F}}{{\partial \theta }} \right]\nonumber\\ &amp; \quad - \frac{1}{{2{\tau _{\textrm{N}}}}}\frac{1}{{\sin \theta }}\frac{\partial }{{\partial \phi }}\left[ \frac{{{\mu _0}{M_{\textrm{S}}}{V_{\textrm{C}}}}}{{\alpha {k_{\textrm{B}}}T}}\left( {\alpha {H_\phi } + {H_\theta }} \right)F\right. \nonumber\\ &amp; \quad \left. + \frac{{2{K_u}{V_{\textrm{C}}}}}{{\alpha {k_{\textrm{B}}}T}}{{\textrm{N}}_\rho }\left( {\alpha {{\textrm{N}}_\phi } + {{\textrm{N}}_\theta }} \right)F \right]\;\nonumber\\ &amp; \quad + \frac{1}{{2{\tau _{\textrm{B}}}}}\frac{1}{{{\textrm{si}}{{\textrm{n}}^2}\vartheta }}\frac{{{\partial ^2}F}}{{\partial {\varphi ^2}}} + \frac{1}{{2{\tau _{\textrm{N}}}}}\frac{1}{{{\textrm{si}}{{\textrm{n}}^2}\theta }}\frac{{{\partial ^2}F}}{{\partial {\phi ^2}}}.\end{align*}

In equation (14), *F* represents the probability density function (PDF) for an ensemble of MNPs and indicates the distribution of ‘spots’ on a unit sphere where the magnetization points. Additionally, ${K_u}$ represents the uniaxial anisotropy constant, ${V_{\text{C}}}$ represents the volume of the magnetic core, ${\tau _{\text{B}}}$ and ${\tau _{\text{N}}}$ are the same time constants as defined in equation (9), $\vartheta $ and $\theta $ are the angles made by the easy axis and moment with *z*-axis respectively while $\varphi $ and $\phi $ are the corresponding in-plane angles. Finally, $U$ and $H$ represent the magnitudes for anisotropy and moment in different directions in the spherical coordinate system.

### Concluding remarks

MNPs have applications in several fields of science and medicine, which makes understanding their behaviors imperative in the field of magnetism. The primary characteristic of MNP is their small size and high surface-to-volume ratio, which leads to a spin canting effect that results in a reduction in *M*_s_ and an increase in *K*_eff_ compared to bulk materials. To model the magnetic behavior of MNPs for real-life applications, different models have been established so far. Researchers attempt to explain the static and dynamic magnetization behaviors of MNPs under various magnetic field conditions. The earliest models such as the SW and Langevin model ignore one or more energy terms and decouple the Brownian and Néel relaxations. Subsequent models treat the relaxations as decoupled, which is practically incorrect. The relaxation models under zero, DC, and slowly varying AC fields assume an equilibrium system and one dominant relaxation process. The non-equilibrium models can remove this limitation but cannot account for the coupling of the two relaxations. The Debye model and the empirical relaxation models are valid for low fields away from saturation. The LLG model is known for capturing magnetization dynamics but ignores Brownian relaxation.

The theory proposed by Rosensweig [17] to decouple the Brownian and Néel relaxations, although making the modeling easier, can result in seriously flawed analyses. In the stochastic Langevin model, the coupled equations of Brownian and Néel motions track both the direction of the easy axis of the MNP and the direction of the magnetization simultaneously. By solving the combined differential equations, more accurate dynamic magnetizations of MNPs can be achieved [15]. However, the convergence criteria are not used regularly either and should be included. The Fokker–Planck model is efficient computationally, but it is less flexible (harder to incorporate other effects), and the series truncation used in the derivation makes it necessary to understand the derivation to use it effectively. The result is that very little work has been done using this Fokker–Planck model. In addition, for most biomedical applications of MNPs, nanoparticles are often used in a clustered form for better MPI imaging performance [18], higher bioassay sensitivity [19], improved hyperthermia performance [20], and more. In these scenarios, MNPs in a cluster are subjected not only to external fields and thermal fluctuations but also to dipole-dipole interactions (i.e. magnetic dipole fields). These interactions should be taken into consideration to more accurately model and predict the collective dynamic magnetization behaviors of MNPs in biomedical applications.

### Acknowledgments

K W acknowledges the financial support from Texas Tech University through HEF New Faculty Startup, NRUF Start Up, and Core Research Support Fund.

## 2. From physical and chemical approaches to biological synthesis routes of MNPs

### Stefano Ciannella^1^, Cristina González-Fernández^1,2^ and Jenifer Gomez-Pastora^1^

^1^ Department of Chemical Engineering, Texas Tech University, Lubbock, TX, United States of America

^2^ Department of Chemical and Biomolecular Engineering, University of Cantabria, Santander, Spain

### Status

**Figure 3.**
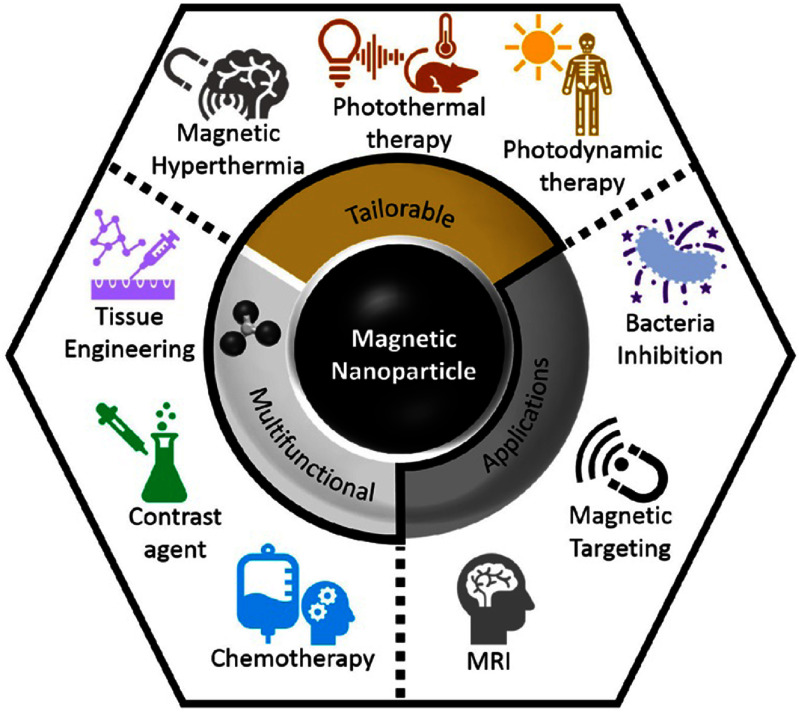
Summary of current applications of MNPs in nanomedicine. Reprinted with permission from [23]. Copyright (2021) American Chemical Society.

The groundbreaking evolution of magnetic nanoparticles (MNPs) in nanomedicine is fundamentally propelled by their unique attributes: a high surface-area-to-volume ratio, chemical stability, dispersibility, magnetophoretic separation capability, low toxicity, and potential for functionalization [21, 22]. MNPs have demonstrated versatility in various biomedical applications [23], including but not limited to MRI, drug delivery, and magnetic hyperthermia for cancer treatment (see figure 3). For these applications, the green synthesis of a variety of nanoparticles has been increasingly investigated as a promising route to mitigate issues associated with conventional physical and chemical methods, such as elevated costs and hazardous byproducts [24, 25]. The historical trajectory of MNP synthesis reflects an ongoing pursuit to enhance their chemical and magnetic properties, as well as investigating cytotoxicity aspects for their medical utility. In this context, synthesis methods play a pivotal role in influencing key tunable features such as shape, size distribution, surface chemistry, and magnetic properties.

The progress in utilizing MNPs in biomedical applications hinges on the techniques employed for synthesizing these nanomaterials. Synthesis methods include physical, chemical, and biological methodologies. Physical methods encompass a wide range of techniques that involve the fractionation of bulk materials (top–down approach) into nanometer-sized particles, with only a few instances of a bottom-up approach in this category (i.e. gas-phase condensation methods). Under the top–down physical methods, ball milling techniques are often employed to produce a variety of nano-sized magnetic and non-magnetic materials. This process unmakes chemical bonds with the kinetic energy of impacting steel balls, resulting in smaller particles with an increase surface-area-to-volume ratio [26]. In contrast, chemical and biological approaches generally adopt bottom–up strategies by assembling atoms/molecules into MNPs [27]. Chemical methods, constituting the majority of currently applied techniques and covered by a vast literature, have proven instrumental in cost-effective MNP production, offering a decent control over shape and size through precise tuning of reaction conditions. Synthetic routes are generally conducted in liquid phase, such as precipitation and co-precipitation techniques, sol–gel, microemulsions, hydrothermal, sonochemical, and polyol methods. For instance, the co-precipitation method, the simplest and oldest chemical approach to MNP synthesis, is based on precipitating precursor materials in a basic aqueous solution. Their resulting physical properties (morphology, size, and dispersion) can be tuned by setting reaction parameters such as the ratio of the precursors, pH, and temperature [28]. Despite their versatility, chemical methods bring along the use of hazardous chemicals in the form of toxic metal precursors, capping agents and stabilizers, along with high temperatures and associated elevated energy comsumption. As an example, chemical synthesis of zinc oxide (ZnO) nanoparticles requires temperatures in the range of 600 °C–1200 °C through different conventional methods [29]. In response to this problem, biological methods have gained increasing attention while being acknowledged as eco-friendly and less energy-intensive alternatives. Biological methods, rooted in living organisms like microorganisms and plants [30], emerge in this context as environmentally friendly alternatives. Based on the biological substrate, the biological synthesis of MNPs can be classified as microorganism-based and plant-based approaches. Microorganism-based synthesis entails the reduction of metal salts to metal nanoparticles by enzymes and can be carried out extracellularly or intracellularly, while the plant-mediated approach is based on the reduction of metal ions to nanoparticles by the bioactive constituents that are present in the plant extract [31]. Biological synthetic approaches have been developed as an effort to develop non-toxic, eco-friendly alternatives for their chemical and physical counterparts. By overcoming drawbacks such as high temperatures, hazardous byproducts, and production costs, biological methods are posed as simple, cost-effective, safe, and environmentally friendly routes for MNP synthesis. Table 1 reports the advantages and disadvantages of several conventional synthesis approaches as well as the main characteristics of the MNPs produced by these manufacturing routes.

**Table 1.** Comparison between several synthesis routes of MNPs [31–35].

Classification	Methods	Advantages	Disadvantages
Chemical	Co-precipitation	Simple, high yield	Bad shape control, wide size distribution
	Sol-gel	Simple, controllable particle size and morphology	Long time, use of toxic solvents
	Microemulsion	Uniform properties, thermodynamically stable	Low yield
	Hydrothermal	Controllable particle size and shape	High pressure and temperature, limitation of reliability and reproducibility

Physical	Ball milling	Large scale production of high purity particles	High energy requirement, long period of milling time
	Laser ablation	Relatively simple	Challenges with prolong time laser ablation
	Ion sputtering	Generation of less impurities than with chemical methods	Challenges with sputtering gas

Biological	Plant-based	Fairly homogeneous particles	Low yield
	Bacteria-based	Abundant bacteria availability	Low yield, bad shape control

### Current and future challenges

The green synthesis of MNPs represents a transformative paradigm, yet several pivotal challenges must be addressed to fully unlock its potential in nanomedicine. The main issue perhaps relies on the lack of standardization and further exploration of optimal process conditions. This advancement should not only facilitate comparisons between studies and promote meta-analysis investigations, therefore improving their assessment toward clinical trials, but also address the problem of reproducibility and consistency of physicochemical properties of greenly synthesized MNPs, which is crucial for medical applications since variations in particle composition and size might, for example, hinder their performance in the context of diagnostics and therapy [32]. For example, through photosynthesis (i.e. plant-based), nanoparticles synthesized from different leaf extracts have shown significant size and shape variability, with recent studies reporting issues related to wide-spread size distributions, structural imperfections, and cluster formation, making it difficult to achieve uniformity and limiting the suitability of green technologies for large-scale manufacturing [36]. Another issue that requires more attention is the slow reaction time, low yield, and conversion rates within biological approaches as presented in table 1, which can potentially turn them less attractive and may affect the economy of the process when compared to various chemical synthesis methods [35]. Additionally, research on mammal toxicity and the accumulation of nanoparticles is still limited: the scarcity of reports providing *in vivo* particle biodistribution and kinetic profiles, essential for regulatory approval, elevates the need for more comprehensive studies. An in-depth exploration of degradation pathways is imperative, given the inadequacy of current *in vitro* assessments [37, 38]. While some studies have demonstrated promising properties and safety attributes of biologically synthesized MNPs, comprehensive *in vivo* assessments, and clinical evaluations are pointed out as necessary for establishing the safety and efficacy of MNPs in medical applications. Furthermore, their interaction with living cells can yield both beneficial and detrimental effects. Limitations such as poor drug loading in drug delivery, overdose risks, non-targeted dispersion causing cellular toxicity, and potential blood coagulation necessitate continuous monitoring and advancements to enhance the functionality of MNPs in nanomedicine applications. Overcoming these constraints requires extensive both *in vitro* and *in vivo* investigations and optimizing synthesis conditions to achieve decent quality and size control while implementing sustainability-driven technologies. The availability of natural materials is another issue that must be addressed to promote new and innovative biological routes to synthesize metal-based nanoparticles from plant extracts. In this direction, a comprehensive toxicological assessment and the optimization of genetically modified microorganisms are crucial for the production of MNPs [36].

### Advances in science and technology to meet challenges

**Figure 4.**
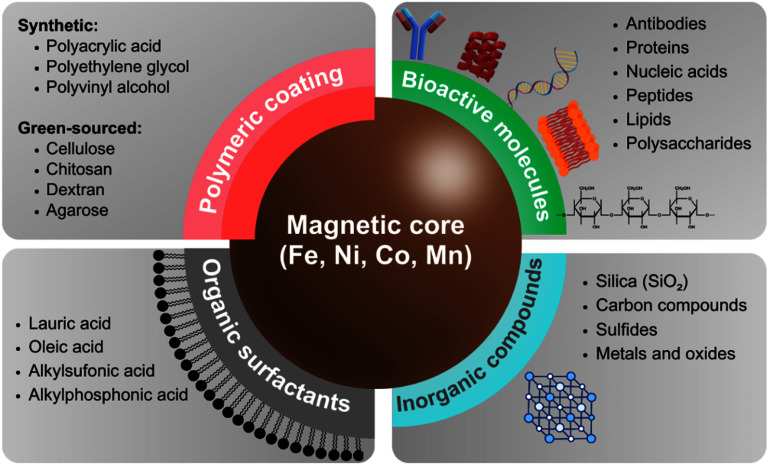
Types of surface coatings commonly used on MNPs and some examples of each sort. Adapted from [33]. CC BY 4.0.

Achieving nanoparticle quality and size control involves advancements in experimental design and characterization techniques. One of the main constraints of green synthesis lies in an inadequate optimization of reaction parameters, or even lack thereof, along with the identification of optimal solvents [37]. In this context, further research efforts are crucial to unravel the intricate impact of different factors on the shape and size of greenly synthesized nanoparticles. These factors include reactant concentration, reaction time, pH, and temperature, to name a few. The understanding of these parameters is essential as they can also influence the polydispersity of the created materials. Additionally, the development of biomaterials derived from plants and microorganisms necessitates further interdisciplinary research combining biology, chemistry, and materials science. Plants and herbs are abundant, safe to handle, and contain numerous primary and secondary water-soluble metabolites such as anthocyanins, tannins, saponins, flavonoids, polyphenols, polypeptides, starches, alkaloids, and terpenoids, which serve as effective chemical reducers and stabilizers, as well as capping agents [24, 36, 39]. Exploring natural resources involves identifying new plant extracts or biological sources (bacteria, fungi, algae, etc) with optimal properties for the green synthesis of MNPs. Associated issues with the production of greenly synthesized MNPs like poor stability, dispersity, and oxidization, which altogether might also curb their applicability in biomedical and therapeutic fields, can be mitigated by including appropriate surface coatings [40]. Figure 4 provides a summary of the most common types of surface coatings used on MNPs to improve their stability and biocompatibility.

Regarding metal nanoparticles, challenges associated with clinical and diagnostic applications demonstrate the need for advancements in benchmarking their physicochemical properties and *in vitro/in vivo* evaluation techniques. Advanced research on surface modification is currently pointed out as a promising approach to broaden the applicability of metal-based MNPs in nanomedicine [41]. In combination with the previously pointed advancement pathways, improved *in vitro* and *in vivo* evaluation methods cannot be stressed enough and are essential for assessing nanotoxicity, therapeutic efficiency, and biodistribution. Furthermore, it is imperative to gain a comprehensive understanding of the biochemical mechanisms underlying the green synthesis of iron-based nanoparticles. While some studies suggest that biologically synthesized nanoparticles exhibit lower toxicity [41], conducting risk assessment studies is crucial to ensure the safety, economic viability, and environmental friendliness of the process.

### Concluding remarks

The green and sustainable synthesis of MNPs is emerging not only as a necessary change of paradigm but also as a cost-effective, safer, and environmentally friendly alternative to conventional chemical and physical synthesis methods, which tend to employ hazardous chemical species and lack an accurate control over shape and size of MNPs, respectively. Various MNP formulations are undergoing clinical testing, suggesting potential acceptance in clinical settings in the near future. However, the current main challenges in their green, sustainable synthesis lie in the standardization of biological synthesis methods, optimization of process conditions, benchmarking physicochemical properties, and comprehensive *in vitro* and *in vivo* toxicological assessments. It is desirable to achieve magnetically active nanoparticles with defined and consistent properties using sustainable and less pollutant methods. Addressing issues such as variability in particle properties and biocompatibility concerns hold the potential to pave the way for MNPs to achieve reliable performance in diagnostics and therapeutics. In this context, surface modification and advanced evaluation methods hold promise for expanding the applicability of MNPs in nanomedicine.

### Acknowledgments

This study was financially supported by Texas Tech University through HEF New Faculty Startup, NRUF Start Up, and Core Research Support Fund. Dr Cristina González-Fernández thanks the Spanish Ministry of Universities for the Margarita Salas postdoctoral fellowship (Grants for the requalification of the Spanish university system for 2021–2023, University of Cantabria), funded by the European Union-NextGenerationEU.

## 3. Surface functionalization on MNPs

### Yuping Bao

Department of Chemical and Biological Engineering, The University of Alabama, Tuscaloosa, AL, United States of America

### Status

**Figure 5.**
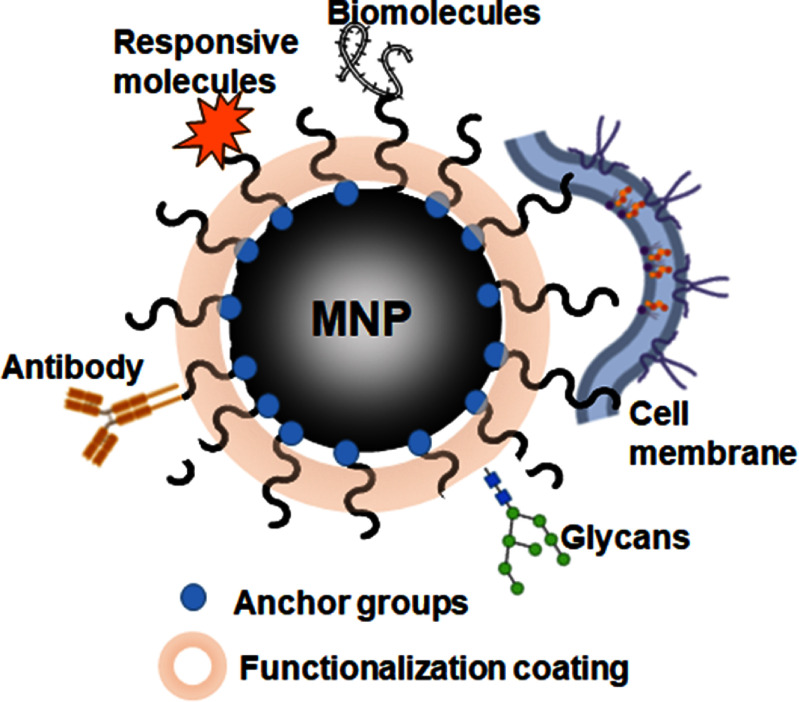
Illustration of functionalized MNPs from various strategies. Original figure prepared by the author.

Surface functionalization is one of the most critical aspects of MNPs that not only directly influence their physical and chemical properties [42] but also define their potential applications [43]. Numerous surface functionalization strategies have been established [44] where the specific factors impacting the magnetic property and application of MNPs were elucidated, including anchor groups on the MNP surface, chain property of the capping molecules, coverage on the MNP surface, extruding functional groups from the MNP surface, and conjugation of specific targeting molecules (see figure 5). Typically, functionalized MNPs are illustrated in figure 5 with various surface functionalities.

Linker chemistry [45] is one of the most common methods for the surface functionalization of MNPs, with carbodiimide (EDC) [46, 47] and N-hydroxyl succinimide (NHS) ester [48] being the most common linkers. The EDC/NHS conjugation links carboxylic and amino groups. Therefore, this technique only applies to molecules with carboxylic and amino groups. In addition, the reaction requires specific conditions, such as acidic conditions (pH 4.5–5.5) for EDC, pH 7.2–8.0 and 4 °C for NHS. Furthermore, the uncontrolled reactions between amine and carboxylic groups lead to concerns about conjugation efficiency and specificity [49, 50]. As for maleimide linker conjugation [51], maleimides form stable thioether bonds with sulfhydryl groups an alkylation reaction. The reaction takes place rather rapidly in the pH range of 6.5–7.5 [52]. However, this liker conjugation only applies to molecules with thiol groups. In addition, at higher pH, maleimide tends to undergo hydrolysis, and interaction with amine groups occurs [52].

Besides linker chemistry, certain surface coating of MNPs can be activated for conjugation such as polydopamine coating. Polydopamine surfaces can be readily activated at basic pH (>8.5) and subsequently react with amine or thiol groups of the conjugating molecules [53, 54].

Additionally, conjugation based on specific molecular recognition is another common strategy, such as enzyme-substrate [55, 56], antibody and antigen [57], and streptavidin-biotin [58]. Here, both enzyme and antibody conjugation are for very specific targets, while streptavidin-biotin is routinely used [59]. Despite the advantage of specificity, biotin-streptavidin interaction requires prior attachment of biotin or streptavidin on MNPs. The biotin-labeled nanoparticles will react with any biotin-binding proteins, reducing the specificity. On the other hand, streptavidin-labeled MNPs have four binding sites on each streptavidin, which leads to uncontrolled attachment of biotin-labeled analyses [60]. In addition, biotin is a natural biological molecule, causing a big concern about the specificity and background effects when involving biotin-rich tissues and extracts.

Recently, surface functionalization of MNPs with biomimetic membranes has become very attractive, including cell membrane [61–64] and extracellular vehicles (EVs) [65]. Here, the cell membrane carries certain properties of the original cells, allowing for desirable functions. Such as tumor targeting for cancer cell membrane-coated nanoparticles [62]. For EVs, MNPs can be loaded into EVs *in situ* during EV generation from cells through MNP treatment or be incorporated post-EV isolation [65, 66]. Compared to cell-membrane coating, the key advantages of EV coatings are cell communication [67] and the ability to cross biological barriers [68].

Beyond colloidal stability against aggregation and biocompatibility, surface functionalization greatly influences the interactions of MNPs with biological systems, such as cellular uptake behaviors for cell-based therapy. For example, the surface capping molecules directly affected cell labeling efficiency and cellular tracking duration [69]. The advancement in the surface functionalization of MNPs has enabled the potential of MNPs as imaging probes, for drug delivery, and theranostic applications. The emerging application of MNPs in cell-based therapy is also moving forward with an increasing number of studies in pre-clinical and clinical settings [70]. Specific surface functionalization strategies of MNPs also made direct cell interactions [71] or *in situ* cell labeling [72] possible. Regardless of the types of applications in nanomedicine, surface functionalization of MNPs has been playing a critical role with well-established protocols to achieve desirable properties. Thus, the surface functionalization of MNPs is evolving into a mature field.

### Current and future challenges

The surface functionalization of MNPs still faces several unmet challenges despite the development of numerous strategies. First, standardized characterization and quantification methods are still lacking. Most of the surface functionalization techniques were validated by the presence of certain functional groups and indirectly quantified by the free ligands pre- and post-functionalization. However, the exact number of capping/targeting molecules is normally not provided. For large targeting molecules (*e.g.* proteins), the orientation of the molecules is also critical for targeting efficiency, which is normally disregarded. The lack of standardized characterization techniques also makes the scalable and reproducible preparation of functionalized MNPs and cross-comparison of similar studies impractical, yielding contradictory results or large discrepancies in findings. Second, *in vivo* immune clearance of MNPs remains an unresolved issue regardless of surface functionalization. The elongated blood circulation has been achieved by using PEGlyation or zwitterionic polymers, but the MNPs still ultimately accumulate dominantly inside immune organs (*e.g.* liver and spleen). Third, surface conjugation strategies have been primarily on using targeting molecules to direct MNPs to bypass biological barriers, such as the blood-brain barrier. However, the efficiency has been inconsistent or insufficient to achieve clinical outcomes. Finally, the long-term stability and potential health risk of functionalized MNPs continue to be a concern from the degradation of the MNP core to the surface capping molecules along with their corresponding degraded small compounds. Therefore, efforts must be continuously made to achieve better quality control of functionalized MNPs, standardized protocols for scalable and reproducible production of functionalized MNPs, and effective quantification with predictable outcomes to ensure clinical translation of functionalized MNPs.

### Advances in science and technology to meet challenges

The advancement in surface functionalization of MNPs has been witnessed by various aspects, including the capability of loading multifunctional drugs for drug delivery, directed localization for imaging and therapy with targeting molecules, stimulus-responsive ligands for on-demand release of cargos, and biomimetic coatings to overcome biological barriers. Particularly, MNPs functionalized with lipid membrane coatings (*e.g.* cell membranes or extracellular vesicles) have demonstrated effectiveness in overcoming issues associated with chemical molecule coatings, such as enhanced tumor targeting and brain delivery. However, the functionalization of MNPs with biomimetic membranes is generally hindered by the reproducible and scalable production of quality membranes. Recently, much attention has been drawn to integrating artificial intelligence and machine learning into surface functionalization of MNPs [73]. These computer-assisted tools have the capability of analyzing massive amounts of available data with the potential of identifying the optimal surface functionalization strategy for a desirable application. Because of the capability of processing a whole body of comprehensive data, the standardization of surface functionalization protocols may become feasible, significantly accelerating the design and implementation of surface functionalization of MNPs. In particular, these computational tools will make the comparison of numerous *in vitro* and *in vivo* studies feasible, generating relevant pharmacological datasets for clinical translation. However, the success of artificial intelligence and machine learning heavily relies on the availability of a large volume of high‐quality data to validate the computational and mathematical models. Valid experimental conditions and system designs are crucial to capture the dynamic nature and complexity of biological systems. Only models that are fully validated with a large amount of quality and relevant data may offer predictive guidance in the new design of surface functionalization. Therefore, the incorporation of artificial intelligence and machine learning methods is anticipated to address some of the current and future challenges, facilitating better and more effective surface functionalization of MNPs and accelerating clinical translation of functionalized MNPs.

### Concluding remarks

The surface functionalization has enabled MNPs’ promises in various areas of nanomedicine, such as drug delivery, cell-based imaging, and therapy, magnetically responsive soft robotics, and lab on a chip. In the last decade, advances have been made with possibilities to achieve enhanced biocompatibility, minimal opsonization, precise targeting, and single-cell sensitivity in imaging and diagnosis. Regardless of *in vivo* or *in vitro* applications, the key limitation for surface functionalization of MNPs is the lack of standardized characterization techniques, qualification strategies, and reproducibility across studies. Much work still needs to be done to fully realize the potential of functionalized MNPs. One promising strategy is the integration of multidisciplinary approaches with the assistance of computational and mathematical models. The future focus needs to be on the reproducibility, scalability, and quantification feasibility of different functionalization approaches, enabling MNPs to reach a level of consistency for clinical or commercial use.

### Acknowledgments

This work was in part supported by NSF-CBET1915873 and the Breast Cancer Research Foundation of Alabama.

## 4. Characterization methods for MNPs

### Jinming Liu^1^, Shuang Liang^2^, Kai Wu^3^ and Jian-Ping Wang^4^

^1^ Western Digital Corporation, San Jose, CA, United States of America

^2^ Department of Chemical Engineering and Materials Science, University of Minnesota, Minneapolis, MN, United States of America

^3^ Department of Electrical and Computer Engineering, Texas Tech University, Lubbock, TX, United States of America

^4^ Department of Electrical and Computer Engineering, University of Minnesota, Minneapolis, MN, United States of America

### Status

Characterization is the first step to evaluate the inherent properties of magnetic nanoparticles (MNPs) to meet the requirements of various biomedical applications [74]. Many characterization techniques have been applied to obtain information on MNPs such as their size and morphology, structure and composition, colloidal stability, magnetic properties, etc. MNPs for biomedical applications are usually composed of an inorganic magnetic core and an organic shell. The size and shape of the magnetic core determine its magnetic properties and applications. Transmission electron microscopy (TEM) is commonly used to characterize the core size, shape, and internal structures. TEM obtains two-dimensional projections of MNPs. Scanning electron microscopy (SEM) is also used to obtain the size, shape, and three-dimensional images. X-ray diffraction (XRD) can estimate the size using Scherrer’s equation. Dynamic light scattering (DLS) is an essential technique to obtain hydrodynamic size and distributions of MNPs in solutions.

Compositions of MNPs are generally characterized by energy-dispersive x-ray spectroscopy (EDS) detecting all elements. Electron energy loss spectra (EELS) is another approach to obtaining elemental compositions, which has a better signal-to-noise ratio, improved spatial and energy resolution, and enhanced sensitivity to elements with lower atomic numbers. X-ray photoelectron spectroscopy (XPS) can provide composition and binding states on the surface of a sample (several nanometres). The structural information of MNPs is critical. For example, even though *α*-Fe_2_O_3_ and *γ*-Fe_2_O_3_ have similar compositions, their magnetic properties are quite different. XRD is a common way to determine structural information. High-resolution TEM (HR-TEM) can also measure structures from images and electron diffractions. Fourier transform-infrared (FT-IR) characterizes bonding between MNPs and organic shells, binding energy, and oxidation states.

Due to the surface canting effect, the magnetic properties of MNPs differ from their bulk materials [27]. Vibrating-sample magnetometer (VSM) is frequently used to characterize the magnetic properties of dried MNPs. Where a sample placed in a coil is subjected to a constant applied field. A voltage signal is generated in the coil by vibrating the sample according to Faraday’s law. Thus, the M-H curves are obtained, which provide information like coercivity, remanence, saturation fields, saturation magnetization, etc. A superconducting quantum interference device (SQUID) is another choice that provides better sensitivity over a VSM.

### Current and future challenges

Characterizing MNPs is crucial for diverse biomedical applications, yet certain characterization techniques pose challenges that require careful consideration. Take TEM as an example, it is a potent method to measure the sizes and shapes of MNPs. Due to the limitation of sample sizes, only a small fraction of samples can be characterized, necessitating considerable effort to secure a truly representative sample for comprehensive TEM analysis. MNPs may generate an external field deflecting TEM’s electron beams, requiring secure sample fixation and the astigmatism of the objective lens needs to be carefully adjusted for clear imaging. High-energy electron beams in TEM may heat up and damage MNPs of interest, especially when characterizing a single nanoparticle. Comparable constraints also extend to other electron microscopic technologies, including SEM. Meanwhile, due to the high vacuum in TEM, dried MNP samples are generally used. More advanced techniques are needed to characterize MNPs in a solution. XRD can only estimate the average size of MNPs according to Scherrer’s equation, lacking size distribution information. If MNPs exhibit an amorphous structure or are extremely small, it becomes challenging to identify a diffraction peak to determine their average size. DLS quantifies the hydrodynamic size distribution of MNPs. It is highly responsive to MNP aggregations, necessitating careful consideration of MNP concentration. The transparency of the DLS media is crucial for optimal light scattering. Additionally, the determination of the average radius of MNPs assumes a perfectly spherical shape, a condition difficult to attain in practical scenarios. Irregularities in the shape of MNPs can lead to measurement errors. The assessment of MNPs’ colloidal stability often involves measuring their Zeta potential. However, obtaining accurate measurements is challenging due to the susceptibility of Zeta potential to various extrinsic factors, such as concentrations and the composition of the solvent.

VSM and SQUID provide bulk magnetic property data for characterization, but individual particles may exhibit variations. Dried MNP samples must be securely fixed on a holder to prevent physical rotation, such as Brownian relaxation. Consequently, VSM and SQUID analyze Néel relaxations in the magnetizations of MNPs under diverse excitation fields.

### Advances in science and technology to meet challenges

Scientists have devoted substantial efforts to addressing characterization challenges. The conventional use of high vacuum in TEM makes direct characterization of MNPs in liquid solutions or biological matrices challenging. Cryogenic TEM (cryo-TEM) has been developed to maintain samples at near liquid nitrogen temperatures, preserving the structure of MNPs in solutions and facilitating TEM imaging [75–77]. Cryo-TEM has proven valuable in observing various phenomena, including the nucleation and growth of particles, the formation mechanism of magnetic meso-crystals, and changes in agglomeration state when particles interact with cells. Mirabello *et al* explored the behavior of Fe_3_O_4_ aggregates using cry-TEM. They observed that ferrous hydroxide precursors, initially with a primary particle size of approximately 5 nm, aggregated into Fe_3_O_4_ primary crystals around 10 nm. The primary crystals then formed orientated and uniform mesocrystals, exhibiting diffraction patterns similar to those of single crystals [75].

Liquid cell TEM is also developed to preserve the liquid state and carry in-situ imaging of liquid phases with particles, which benefits the understanding of how particles perform in a liquid phase [78, 79]. Liquid samples are sealed in a small cell, which is generally made by microelectronic fabrication on silicon wafers. Samples are sealed by electron-transparent windows, such as SiN. Graphene and thin amorphous carbon film have also been used to preserve volatile samples like biological cells, under the high vacuum in TEM. Liquid cell TEM has many applications such as the in-situ growth of nanoparticles (e.g. Cu, Pt, iron oxyhydroxide, etc), core@shell structures (e.g. Au@Pd, Fe_3_Pt@Fe_2_O_3_, PtNi@Ni, etc), and the movement and interaction of nanoparticles. Powers *et al* studied the dynamic interaction of Pt-Fe nanoparticles during the assembly process using a liquid cell TEM [80]. Initially, 2D loosely packed nanoparticles were observed, which then transformed into 1D chains, and eventually formed 2D lattices over time.

Scanning SQUID biosusceptometry is developed to detect MNPs with low concentration in both *ex vivo* and *in vivo* [81]. It has a pickup coil close to the samples. The signal from a pickup coil is amplified and transferred to a SQUID sensor. A sub-millimeter spatial sensitivity could be achieved when detecting MNP-labeled tumors. MNP imaging in rat liver and heart in both *in vivo* and *ex vivo* was reported. A 0.1 attomole sensitivity was also demonstrated by integrating a microfluidic array into a scanning SQUID. Even a single MNP can be detected [82].

A recent technique, magnetic particle spectroscopy (MPS), has emerged for characterizing the magnetization dynamics of MNPs in liquid, encompassing coupled Brownian and Néel relaxations [83]. This MPS technique is used for evaluating the suitability of MNPs as tracers for MPI applications. In the MPS characterization process, an ensemble of MNPs suspended in a solution is subjected to a sinusoidal magnetic field, also known as the drive field or excitation field, with a frequency denoted as *f*. This field periodically saturates the magnetizations of the MNPs, generating higher harmonics at 3*f*, 5*f*, 7*f*, and so forth, due to the nonlinear responses of their magnetizations. These higher odd harmonics result from coupled Brownian and Néel relaxations, and their manifestation is mitigated by thermal fluctuations. The intrinsic magnetic properties of the MNPs, including relaxation time [84], saturation magnetization, anisotropy, magnetic core size, hydrodynamic size, as well as the viscosity and temperature of the solution [85], influence the characteristics of these higher harmonics.

### Concluding remarks

This section presented basic characterization techniques of MNPs for biomedical applications. One may pick up the right techniques to best describe MNPs for specific applications. Essential techniques need to be considered such as representative samples for TEM to obtain size, shape, composition, and structural information on a sufficient number of MNPs. As there are challenges and limitations of some characterization techniques, several techniques may be used together to get comprehensive information. For example, XRD only provides average size information of MNPs, but TEM can help obtain size distribution, and DLS provides the hydrodynamic radius of MNPs. By combining all this information, one would have a more comprehensive idea about the size and size distribution of MNPs. It will also help in understanding other properties. Several advanced techniques aimed at addressing the challenges should also be considered as options to obtain more comprehensive information on samples.

### Acknowledgments

K W acknowledges the financial support from Texas Tech University through HEF New Faculty Startup, NRUF Start Up, and Core Research Support Fund. J-P W acknowledges the financial support from the Institute of Engineering in Medicine and the Robert F Hartmann Endowed Chair professorship by the University of Minnesota.

## 5. Magnetic separation for sample enrichment

### Xian Wu^1^, Linh Nguyen T Tran^2^, Karla Mercedes Paz González^2^, Hyeon Choe^1^, Jacob Strayer^1^, Poornima Ramesh Iyer^1^, Jeffrey Chalmers^1^ and Jenifer Gomez-Pastora^2^

^1^ William G Lowrie Department of Chemical and Biomolecular Engineering, The Ohio State University, Columbus, OH, United States of America

^2^ Department of Chemical Engineering, Texas Tech University, Lubbock, TX, United States of America

### Status

Magnetic separation has emerged as an advanced technology, gaining increasing attention in recent years within the fields of biomedicine and biology. The separation of biological materials from biofluids, such as uncommon cellular entities, pathogens, and other biomolecules, plays a pivotal role in diverse biomedical fields, including diagnostic modalities, therapeutic interventions, and foundational investigations in cellular biology. Comprehending the complexities associated with cellular dynamics often requires extracting and enriching distinct cell subsets and/or their components before their analysis, aiming to mitigate the inherent diversity within the scrutinized specimen. Instances of cell populations of interest involve but are not limited to, stem cells, circulating tumor cells (CTCs), cancer stem cells, and subpopulations of white blood cells (WBCs). The magnetic enrichment process of these cell types encompasses either the utilization of magnetic nanoparticles (MNPs) as magnetic labels for the separation of diamagnetic (non-magnetic) cells or label-free systems for the separation of paramagnetic cell types. Labeled magnetic separations commonly involve the deployment of MNPs, such as magnetite or maghemite, within the size range of 1–100 nm [86, 87]. These MNPs exhibit distinctive physical and chemical characteristics, augmented magnetization, and recent advances in MNP synthesis facilitate the binding of functionalizable ligands to their surface. Additionally, under small sizes, their superparamagnetic attributes enable facile manipulation through external magnetic fields. Unlike traditional labeled-based methodologies, there is a growing interest in label-free separation approaches that aspire to isolate disease-related entities without the necessity of supplementary labeling agents [88]. These label-free methodologies capitalize on the target entities’ inherent magnetic properties to achieve specific and efficient separation.

### Current and future challenges

**Figure 6.**
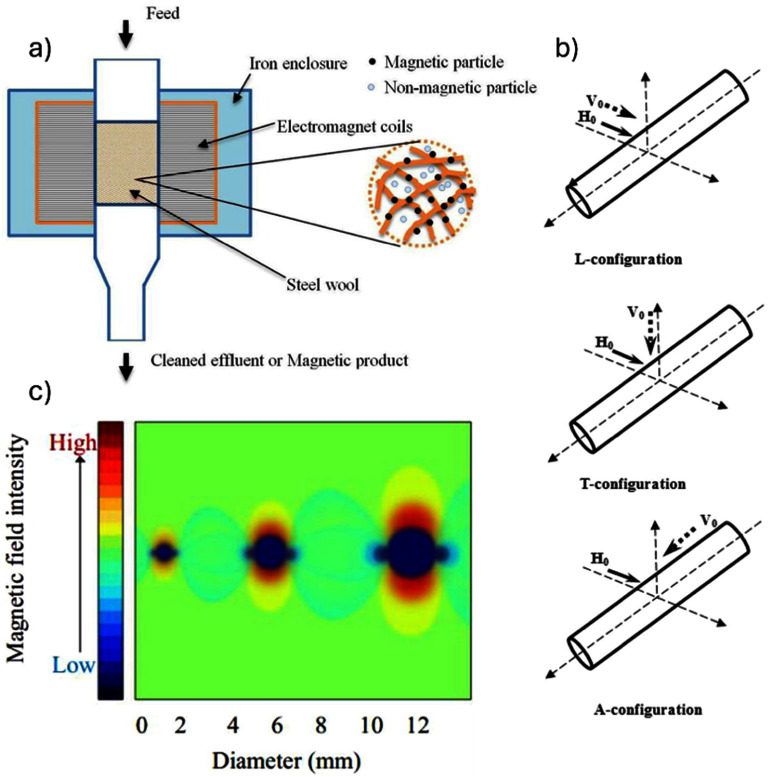
(a) Schematic diagram of the HGMS process. (b) Three possible configurations of the rod matrix in HGMS systems. (c) Effect of the rod diameter on the magnetic field distribution in HGMS matrices. Reprinted from [92], Copyright (2017), with permission from Elsevier.

Magnetic enrichment is a complex process influenced by various factors and forces that govern the behavior of materials when subjected to an external magnetic field. These forces encompass the magnetic force, gravitational force, viscous drag, buoyancy, inertia, particle-fluid interaction, Brownian motion, as well as inter-particle phenomena that include magnetic dipole interactions and Van der Waals forces [89]. The application of magnetophoresis has proven to be effective in driving sample enrichment of several bio-entities, utilizing both high gradient magnetic separation (HGMS) columns and low gradient magnetic separation (LGMS) systems [90]. HGMS typically involves the use of packed columns of ferromagnetic materials to capture specific entities (figure 6(a)). The column packing comprises wires with diameters of approximately 50 *μ*m, with gap distances ranging from 10 *μ*m to 100 *μ*m (figure 6(b)) [89]. Once the column is magnetized by an external magnetic field, the target entities (labeled cells or paramagnetic biomaterials) flow through the column and get effectively trapped on the packing surface. HGMS offers advantages, such as the ability to generate exceptionally high magnetic gradients, resulting in elevated magnetic forces acting on the entities (figure 6(c)). However, some existing drawbacks include the generation of inhomogeneous magnetic fields and gradients due to the irregular packing material, which complicates the description of the process. In addition, HGMS may lack specificity in capturing only the magnetic particles or cells, leading to potential contamination from non-magnetic entities inadvertently trapped by the magnetic matrix. The most well-known HGMS system for cell separation is the MACS (Magnetic Activated Cell Sorting) developed by Miltenyi Biotec [91].

**Figure 7.**
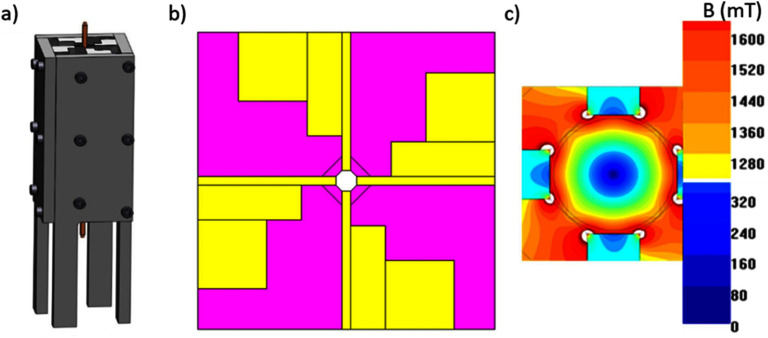
(a) Quadrupole magnetic sorter for magnetic particle separation. (b) Top view of the steel pole pieces (purple) and NdFeB magnets (yellow). (c) Cross-sectional field map. Reprinted from [98], Copyright (2020), with permission from Elsevier.

To overcome the limitations of HGMS, research efforts in magnetic separation have focused on the development of LGMS [90]. LGMS provides a cost-effective alternative capable of achieving separation of target entities using external magnetic field gradients typically on the order of <100 T m^−1^. An LGMS setup includes one permanent magnet or an arrangement of them to provide the magnetic fields and gradients required for separation. Unlike HGMS, LGMS does not require specialized, pre-packed columns, significantly reducing installation and operational costs, minimizing contamination due to mechanical filtration of non-magnetic particles/biomaterial, and allowing the separation process to be continuous [93]. LGMS systems utilize permanent magnet arrays to generate stable and reproducible magnetic gradients, allowing for simpler setups, easier description, modeling, and scale-up of separation processes. For large-scale processes (generally within water treatment applications), the operational cost of LGMS is notably lower, by approximately four-fold as compared to HGMS, $0.13/m^3^ of sample treated versus $0.52/m^3^ respectively, due to lower energy requirements and the absence of complex electromagnets and matrices [94]. Despite its cost-effectiveness, LGMS faces serious challenges in the separation of small particles or weak magnetic materials due to the low magnetic field gradients generated in the system, rendering it unable to isolate entities in a reasonable amount of time. Indeed, the process often requires extended periods of magnetic exposure, ranging from several hours to days, depending on entity size and concentration [95]. Nevertheless, the development of novel devices based on quadrupole magnet arrangements has shown to be promising for the separation and magnetic enrichment of small nanosized particles in a matter of minutes [96–98] and paramagnetic cells and biomolecules in a continuous-flow operation mode [99]. Figure 7(a) presents a quadrupole magnetic sorter that uses permanent magnets to separate and enrich magnetic materials.

### Advances in science and technology to meet challenges

In the pursuit of increasing the separation efficacy and expanding the applicability of HGMS systems, significant investigations have focused on the optimization of matrices, packing materials, and geometries within these separators (figure 6(b)). The fabrication of magnetic matrix/packing materials has involved the utilization of substances characterized by heightened permeability, augmented corrosion resistance, and superior abrasion resistance [92]. Additionally, the optimization of packing geometries has been systematically undertaken to mitigate fluidic impedance and amplify the capture area per unit volume, thereby enhancing the overall collection efficiency. Moreover, labeled separations have been and continue to be optimized by developing novel MNPs. This optimization includes engineering the material composition to augment saturation magnetization, consequently elevating both the magnetic force acting on the material as well as the local field gradient generated by the MNPs. Furthermore, in magnetophoresis, the geometry and arrangement of magnets influence the magnetic field gradient and its distribution within the separator volume, which are pivotal factors for improving the device’s performance. As shown in figure 7(b), a quadrupole geometry involving the arrangement of four permanent magnets has been employed to establish regions with extremely high field gradients in the order of >1000 T m^−1^ within the device. Finally, numerical simulations have proven to be very useful to comprehensively understand the inherent mechanisms of magnetophoresis and important parameters governing the magnetic separation process. Recent works have addressed the utility of numerical models to design and optimize magnetophoretic devices [100]. These models comprise a set of equations that describe the forces acting on magnetic materials (particles, cells, magnetically labeled biomaterials), mainly the magnetic force and fluidic drag force, and predict the magnetic material motion within the device as a function of the balance of these forces. These computational models are crafted to design separation devices and facilitate the exploration of pertinent process parameters, thus performing process optimization. Additionally, these numerical models can be used to explain experimental phenomena, thereby helping the experimental design [101]. Notably, the simulations can be employed to study the separation process within the device, encompassing predictions regarding the trajectory of the material and the capturing regions inside the separator [102]. Furthermore, the simulations are instrumental in analyzing the impact of factors such as magnetic field gradient (figure 7(c)), MNP size and concentration, sample flow rate, and enrichment performance.

### Concluding remarks

Magnetic separation tools have garnered growing interest across diverse domains, particularly in sample enrichment applications in biomedical fields. Indeed, this technology is particularly useful for rare cell isolation processes, such as the recovery and analysis of cancer cells for early diagnosis. Magnetic separation methods can be broadly classified into two categories: labeled-based techniques and label-free magnetophoresis. Labeled magnetic separation primarily involves utilizing MNPs, such as magnetite or maghemite, to confer magnetic properties to the target entity (a diamagnetic material). Concurrently, there is a rising interest in the label-free separation of biological materials, aiming to enrich samples based on their weak paramagnetic moments without relying on additional labeling agents. Nevertheless, these technologies face several challenges that need to be overcome to exploit the full potential of magnetophoresis. In this chapter, we have highlighted the most critical issues that both high-gradient and low-gradient magnetic separation filters need to solve to increase separation performance (recovery and purity) and throughput, as well as recent advances in the field of magnetic separation.

### Acknowledgments

This research was funded by Texas Tech University through HEF New Faculty Startup, NRUF Startup, and Core Research Support Fund. We also wish to thank the National Heart, Lung, and Blood Institute (1R01HL131720-01A1) and DARPA (BAA07-21) for financial assistance.

The authors wish to thank Dr Ioannis H Karampelas for their valuable contributions and input to this contribution of the roadmap.

## 6. MNP-based bioassays

### Vinit Kumar Chugh^1^, Shuang Liang^2^, Kai Wu^3^ and Jian-Ping Wang^1^

^1^ Department of Electrical and Computer Engineering, University of Minnesota, Minneapolis, MN, United States of America

^2^ Department of Chemical Engineering and Materials Science, University of Minnesota, Minneapolis, MN, United States of America

^3^ Department of Electrical and Computer Engineering, Texas Tech University, Lubbock, TX, United States of America

### Status

Given the nonmagnetic properties of most biological samples, using magnetic nanoparticles (MNPs) as labels for detecting biological target analytes provides the inherent benefit of minimal background noise and, consequently, improved detection limits compared to similar chemical or optical labels. This distinctive characteristic has rendered MNPs a focal point of extensive utilization and investigation within the realm of detection.

**Figure 8.**
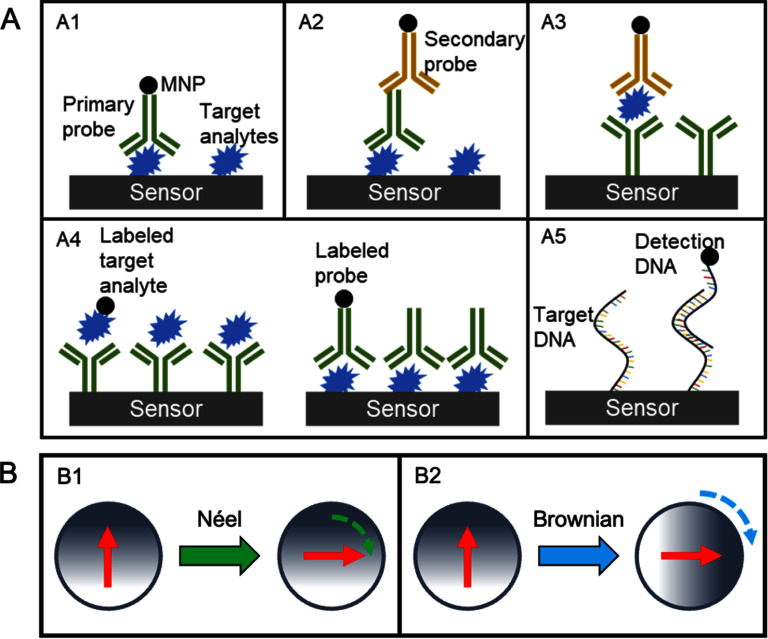
(A) Schematic drawing of various MNP-based bioassays. (A1) Direct assay. (A2) Indirect assay. (A3) Sandwich assay. (A4) Compactivity assay: limited number of probes (left) and limited number of target analytes (right). (A5) DNA assay. (B) Schematic representations of (B1) Néel relaxation and (B2) Brownian relaxation. Original figure prepared by the authors.

To specifically identify and establish a quantitative relationship between biological target analytes and MNP labels, distinct bioassays based on MNPs have been developed. Depending on the nature of the target analytes, the bioassays can be categorized into protein-based and DNA-based bioassays. The protein-based assays can further be divided into (a) direct bioassay, (b) indirect bioassay, (c) sandwich bioassay, and (d) competitive bioassay. In the case of a direct bioassay approach (figure 8(A1)), target analytes bind directly to MNP-labeled detection probes. This method is simple but less sensitive thus suitable only for abundant molecules. For increased sensitivity, an indirect bioassay approach is used (figure 8(A2)), where target analytes bind to primary detection probes, and these probes are then attached to MNP-labeled secondary probes. The sandwich assays (figure 8(A3)) further improve sensitivity by employing a capture probe on the device’s surface, binding to target analytes, which then attach to MNP-labeled detection probes. However, this method requires at least two binding sites on the target analytes, limiting its use for small molecule detection. Alternatively, the competitive bioassay approach (figure 8(A4)) is utilized, where labeled and unlabeled probes or target analytes compete for limited binding sites. When the target analytes are DNA fragments, a DNA-based bioassay can be established by immobilizing probe DNA on the sensor’s surface. Subsequently, the probe DNA can bind to MNP-labeled target DNA fragments (figure 8(A5)). In one example, de Olazarra *et al* [103] demonstrated the establishment of a DNA-based bioassay using MNPs as labels on giant magnetoresistance (GMR) biosensors for gene expression diagnostics. Initially, mRNA was isolated and reverse-transcribed to produce cDNA. Then a PCR reaction generated biotinylated amplicons, followed by denaturation to obtain single-stranded DNA (ssDNA) as the target. The ssDNA was then hybridized with a complementary probe immobilized on the GMR sensor surface. Post-hybridization, streptavidin-coated MNPs were bound to the biotinylated ssDNA, facilitating quantification of the target DNA.

The MNP-based bioassays find applications in both surface-based and volumetric testing environments. Surface-based implementations employ a functionalized surface for specific capture of target analytes and MNP labels for quantitative assays. Under the application of an external magnetic field, magnetization response from the captured MNPs is generated following the Néel relaxation mechanism (figure 8(B1)) which can be detected by different magnetic sensor implementations. Prominent sensors utilized for quantification of MNP labels in surface-based bioassays include (a) magnetoresistive (MR) sensors [104–106], (b) Hall-effect sensors [107], (c) giant-magnetic impedance (GMI) sensors [108], and surface-based magnetic spectrometers [109]. In the volumetric bioassay process, MNPs coated with capture ligands are utilized. Upon addition of a test sample constituting target analytes, these MNPs undergo cluster formation through ligand affinity. MPS is one of the prominent methods for volumetric MNP-based bioassay implementation [110]. MPS relies on the Brownian relaxation of MNPs (figure 8(B2)) under the external magnetic field which is dependent on the hydrodynamic size of the MNPs. Cluster formation following the addition of analytes leads to a change in the Brownian relaxation of MNPs and thus their magnetization response which can be captured for a quantitative analysis of analyte presence. In one such example, Wu *et al* utilized the volumetric MPS approach for the detection of SARS-CoV-2 spike and nucleocapsid protein detection [111]. The Brownian relaxation-dominated MNPs were surface functionalized with specific capture antibodies which allowed for MNP cluster formation upon addition of respective analytes. The formation of clusters upon specific binding leads to a change in the Brownian relaxation of MNPs causing a reduction in their magnetization response and thus the MPS harmonic signal. By utilizing Brownian-dominated MPS detection methodology, authors reported sensitivities of 1.56 nM and 12.5 nM for SARS-CoV-2 spike and nucleocapsid proteins respectively. The other popular mechanism for volumetric bioassay is based on nuclear magnetic resonance (NMR) phenomena. In NMR, MNP cluster formulation modulates the transverse spin relaxation time (T_2_) for the surrounding water molecules and thus quantifies the presence of analytes in a test volume [112].

### Current and future challenges

Surface-based detection methods typically exhibit higher sensitivity compared to volumetric detection methods. Depending upon the magnetic sensor implementation, an improved sensitivity is mainly because of two reasons: (1) the reaction surface works as a concentrator for analytes and MNPs thus, allowing for improved sensitivity, and (2) the proximity of MNPs to the magnetic sensor is significantly closer thus, leading to a higher signal per magnetic label. However, there are significant challenges associated with this sensing topology. Firstly, the surface-based assays require multiple wash steps to remove unbound analytes, probes, and MNPs thus making the assays significantly complex and time-consuming for a layperson to operate. Secondly, for the chip-based assays, cost-per-assay tends to be higher as specialized tooling and fabrication are needed for the creation of single-use magnetic sensors as their surface is functionalized for the assay mechanism. Thirdly, due to the rapid decay of the magnetic field generated by the MNPs with distance, surface-based detection methods are generally less effective for detecting large target analytes as the MNPs are immobilized further away from the sensor surface.

On the other hand, MNP-based volumetric assays offer fast and one-step implementations, making them better suited for use in point-of-care settings. The cost per test is also lower as there is no need for specialized single-use sensor systems. However, this methodology faces significant challenges regarding specificity, signal-to-noise ratio (SNR), sample preparation, and assay standardization. Volumetric assays mostly rely on the use of polyclonal capture antibodies coated MNPs. On one hand, this facilitates better clustering and provides improved assay sensitivity, there is also an unintended setback in the specificity of the assay as polyclonal antibodies have a higher probability of binding to non-specific similar targets leading to false positive results. Similarly, background noise from unbound MNPs leads to poorer SNR. Although volumetric assays are easier to operate when handling complex samples such as blood, cellular uptake of MNPs can lead to false positive results. Lastly, the dependence of MNP magnetization response on the physical characteristics of the test sample such as viscosity and temperature leads to standardization concerns in realizing quantitative assay implementation.

All MNP-based biosensors rely on detecting the magnetic signals from MNP labels. The signal is proportional to the number of target biomarkers in the sample, realizing quantitative bioassay purposes. Thus, MNP properties such as variations in MNP size, shape, and magnetic moment will affect the assay results.

### Advances in science and technology to meet challenges

Recently, notable advancements have been made to address the need for ease of use and lowering time consumption in surface-based assays by integrating microfluidics into their devices. The significant advantages of this effort include (1) increased sensitivity by confinement of samples to smaller volumes, (2) shorter analysis time as the laminar and turbulent flows have been explored for achieving faster binding of reagents and (3) improved automation to carry out sample addition and wash steps thus making the systems user-friendly and easier to operate by a layperson. The challenge regarding cost-per-assay for the chip-based assays has also been helped by the incorporation of better fabrication systems for achieving higher sensor yield per silicon wafer and thus minimizing the cost for the single-use sensors.

For the MNP-based volumetric assays, sample pre-treatment and enrichment have been adopted to address the challenges of sensitivity, SNR, and sample standardization. The pre-treatment steps include filtration of complex biological samples such as saliva and blood to remove debris and large cells to avoid false positives through particle aggregation. The enrichment step is usually implemented following cluster formation of MNPs upon analyte addition. This may include filtration for removal of un-bound MNPs for improved SNR performance, and magnetic separation to concentrate MNP-bound analytes in a smaller region to improve sensitivity. Different ways of sample enrichment have been better discussed in section 5 of this roadmap. The standardization issues about sample viscosity variations are also addressed by dispersing the enriched samples in known media. The sensitivity improvements for volumetric assays have been further explored by modulating the relative concentration of MNPs to analytes present in test samples.

**Figure 9.**
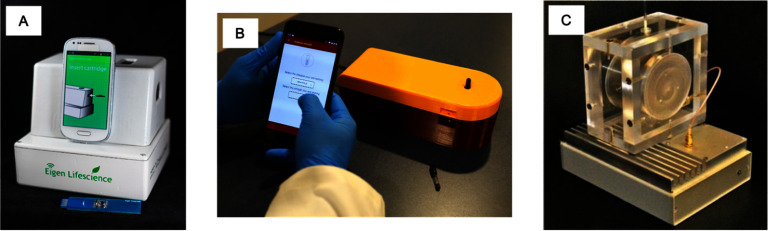
Point-of-care devices developed utilizing magnetic detection of MNPs using (A) Giant magnetoresistance (GMR) sensor. (B) MPS sensor. (C) NMR sensing implementation. (A) Reprinted from [113], Copyright (2016), with permission from Elsevier. (B) Reprinted with permission from [114]. Copyright (2021) American Chemical Society. (C) From [115]. Reprinted with permission from AAAS.

For synthesizing high-quality MNPs, various methods are in development including physical, chemical, and biological approaches. Chemical methods, such as coprecipitation, hydrothermal, or sol-gel synthesis, are becoming the preferred approach due to their ability to precisely control particle size, shape, and distribution [74]. However, significant efforts are still needed to produce biocompatible MNPs in a single step. Furthermore, the inclusion of these recent advancements has paved the way for the creation of point-of-care devices utilizing MNPs and magnetic sensing approaches for use in resource-limited geographies (figure 9).

### Concluding remarks

MNP labels offer several advantages over fluorescent labels for bioassay applications. MNP-based bioassays provide better sensitivity due to their high magnetic moment and improved SNR performance owing to the inherent minimal background noise. Fluorescent labels lose their fluorescent intensity over time due to photobleaching whereas the magnetic properties of MNPs do not significantly decay making them better shelf stable. MNPs can also be easily coated with biocompatible materials making them better suited for biological applications which is not always the case for the fluorescent labels that can sometimes be toxic. Utilization of magnetic manipulation techniques for isolation, separation, and enrichment of target analytes makes the MNP-based assays better suited for high sensitivity and specificity applications. Lastly, MNPs can be detected without additional labeling reagents, thus simplifying the assay procedure and reducing cost-per-assay. With these inherent advantages, several exciting future trends are emerging for MNP-based bioassays on multiplexing and point-of-care diagnostic applications. MNPs with different magnetic properties can be used for the simultaneous detection of multiple analytes in a single bioassay enabling high throughput screening. Their high sensitivity, better SNR and label-free detection capabilities along with integration with microfluidics make them well-suited for enabling fast and automated point-of-care detection in resource-limited settings.

### Acknowledgments

This study was financially supported by the Institute of Engineering in Medicine, the Robert F. Hartmann Endowed Chair professorship, the U.S. Department of Agriculture-National Institute of Food and Agriculture (NIFA) under Award Number 2020-67021-31956. K W acknowledges the financial support by Texas Tech University through HEF New Faculty Startup, NRUF Start Up, and Core Research Support Fund. J-P W acknowledges the financial support from the Institute of Engineering in Medicine and the Robert F Hartmann Endowed Chair professorship by the University of Minnesota.

## 7. MNP-based medical imaging: magnetic resonance imaging (MRI)

### Bahareh Rezaei, Shahriar Mostufa and Kai Wu

Department of Electrical and Computer Engineering, Texas Tech University, Lubbock, TX, United States of America

### Status

**Figure 10.**
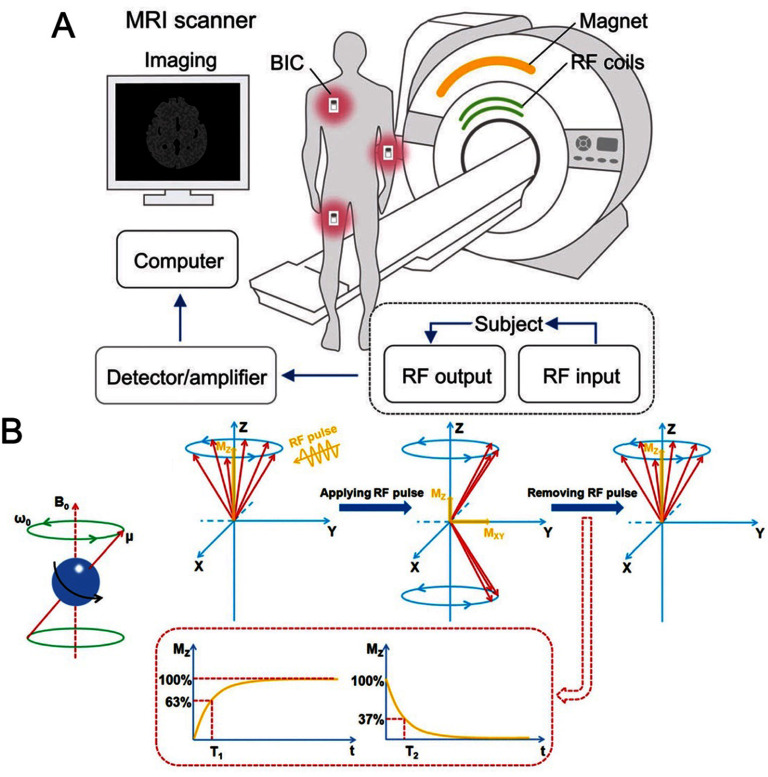
Schematic views of (A) an MRI scanner and (B) T1 and T2 relaxations. (A) Reproduced from [116]. CC BY 4.0. (B) Reproduced from [117] with permission from the Royal Society of Chemistry.

MRI has gained popularity as a non-invasive clinical imaging technique for the diagnosis and/or staging of human illnesses. The technique of producing imaging contrast with MRI is based on the intrinsic magnetic characteristics of human tissues. The method generates images by utilizing radiofrequency energy that the tissue being photographed absorbs and emits. Typically, MRI needs this situation to create medical images of the body (see figure 10(A)): (1) a static magnetic field, B_0_, which is aligned either parallel or antiparallel to the *Z* direction; (2) application of ‘transmit’ RF magnetic fields to excite the nuclei, and (3) passive conductors or ‘receive coils’ near the sample to detect the signal.

When subjected to an external static magnetic field and a radiofrequency magnetic field, protons realign themselves again in an equilibrium state (low energy state), which is the direction of the main magnetic field. As the RF pulse is turned off, two distinct processes lead to relaxation: longitudinal relaxation times (T1) and transverse relaxation times (T2) (see figure 10(B)). The T1 and T2 are useful parameters for comprehending the dynamics of water molecules in biological systems. MRI contrast between diseased and normal tissue is frequently determined by T1 and T2 relaxation periods that differ due to variations in tissue microstructure.

Until a few decades ago, the detection of soft tissue tumors usually did not take place unless in the late stage of the illness. Before CT and ultrasound imaging were developed, radiologists essentially did not know soft tissue diseases or malignancies. Unfortunately, these methods suffered from inherent drawbacks, such as the poor specificity of ultrasound and the poor contrast resolution of the CT. However, these limitations were solved by the MRI method due to its superior soft tissue contrast resolution. MRI does not involve radiation exposure like CT and does not depend on the operator’s skill as much as ultrasound imaging. Unlike PET, MRI provides detailed anatomical and structural information about tissues and organs. Soft tissue tumors, like kidney, breast, ovarian, etc, are imaged at an early stage of the disease with the help of the MRI method. It can be said that MRI is broadly useful to image the central nervous system to assess cardiovascular diseases such as congenital heart disease, myocardial infarction (MI), dilated cardiomyopathy, and large-vessel disease [118]. Furthermore, employing qualitative T1 and T2 weighted imaging, the anatomical location and morphological characteristics of brain tumors and metastases have been comprehensively identified [119].

### Current and future challenges

Although MRI is one of the most significant imaging modalities in the clinic, its inability to detect disease lesions or structural changes accurately due to inherent low sensitivity continues to be a barrier. To distinguish biological targets from normal tissue, MRI contrast agents that can reduce the longitudinal (T1) or transverse (T2) relaxation durations of water protons in the region of interest are frequently utilized. Gadolinium (Gd) complexes as T1-weighted MRI contrast agents are commonly utilized in clinical settings. The long electron spin relaxation period and seven unpaired electrons of Gd^3+^ can effectively induce the longitudinal relaxation of hydrogen [120]. However, severe negative effects of Gd-based contrast agents (GBCAs) have been reported despite their effectiveness in improving MRI contrast. It has been demonstrated that GBCAs can result in lethal Nephrogenic Systemic Fibrosis (NSF), which can induce fibrotic skin contractures and, in severe cases, shattered bones or even death [121]. Apart from the risk of NSF, Gd deposition in the brain is the issue that is now receiving the most attention because numerous brain regions have also been identified to contain Gd [122]. Also, brain lesions are worse in those with liver or kidney issues due to their lower body capacities to eliminate GBCAs. While studies have indicated that the dentate nucleus (DN) and globus pallidus (GP) contain the largest quantities of Gd, other tissues including the frontal lobe cortex and cerebellar and frontal lobe white matter also contain Gd [123]. Another important risk factor is the presence of more Gd in bones than in other tissues. After GBCA injection, Gd can linger in the bones for almost 8 yr. It can also serve as a Gd reservoir and take the place of calcium in the bone matrix [124].

**Figure 11.**
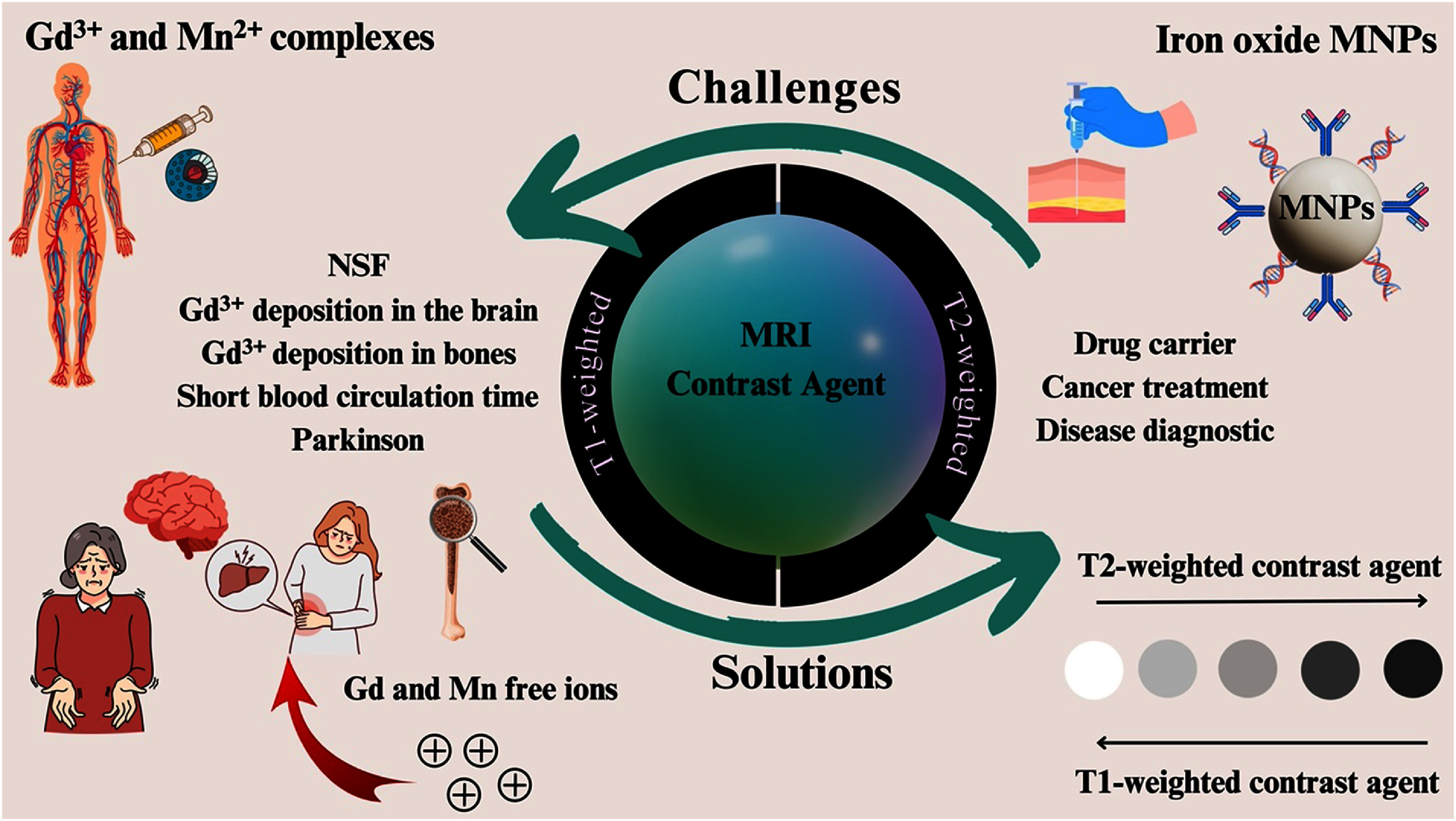
Challenges and opportunities of different MRI contrast agents. Original figure prepared by the authors.

Thus, researchers have focused on using Mn^2+^ as a T1-weighted MRI contrast agent. Since Mn^2+^ has a very short plasma half-life in its ionic state, it is not thought to be a very useful contrast agent. It is usually entrapped in a liposomal formulation for MRI application. However, manganese (Mn) itself is still problematic in terms of toxicity, though. When the body is exposed to high concentrations of Mn, the brain is especially sensitive. This may result in ‘manganism,’ a neurodegenerative condition with symptoms resembling those of Parkinson’s disease (see figure 11) [125]. Manganism is associated with neurochemical changes such as reduced protein aggregation, excitotoxicity, iron-homeostasis, mitochondrial dysfunction, oxidative stress, and altered homeostasis. These alterations have been connected to dopaminergic neuron degeneration [126].

### Advances in science and technology to meet challenges

The growing concern about Gd and Mn complexes as MRI contrast agents has led to increased research on various nanosized biocompatible materials for *in vivo* applications. Iron-oxide-based nanoparticles (IONPs) stand out due to their ease of formulation, functionalization, biocompatibility, and cost-effectiveness. Besides their role as contrast agents, these nanoparticles serve as drug carriers in treating a wide range of diseases like cancer, cardiovascular issues, neurological disorders, autoimmune conditions, and infectious diseases [127]. When iron oxide nanoparticles produce T2-weighted MR images, susceptibility artifacts distort the background image, and the resulting dark signal can occasionally confuse the clinical diagnosis because it can be mistaken for signals from bleeding, calcification, or metal deposits [128]. Consequently, the development of new classes of MR contrast agents that possess T1/T2, or dual-contrast capacity is urgently needed (see figure 11) [129]. In 1996, the Food and Drug Administration (FDA) authorized the first iron oxide nanoparticle product, which had a hydrodynamic size range of 40–150 nm. Under the commercial names ferumoxide (Endorem^®^/Ferridex^®^) and ferucarbotran (Resovist^®^), these standard superparamagnetic iron oxide nanoparticles (SPIONs), coated with dextran, were designed for liver imaging. A prominent side effect of Ferridex^®^ was acute back pain following injection; this resulted in the drug’s 2011 market withdrawal, while Resovist^®^ is still accessible in certain countries, such as Japan. The year 2011 saw the introduction of more medications such as ferumoxtran (Combidex^®^), originally intended for the imaging of lymph node metastases of prostate cancer, and ferumoxytol (Feraheme^®^), intended for the treatment of iron deficiency anemia in patients with chronic kidney disease (CKD). Due to their small core sizes, these latest agents facilitate the advancement of ultrasmall superparamagnetic nanoparticles (USPIO) in the 20–40 nm range. Ferumoxytol is still used for its intended purpose and off-label as an MRI angiography agent in patients unable to use Gd, even after the FDA issued a black box warning for the drug due to serious hypersensitivity responses. Furthermore, formulations used as oral gastrointestinal contrast agents are worth mentioning as well. Examples are ferristene (Abdoscan^®^) and ferumoxsil (marketed as GastroMARK^®^ in the EU and Lumirem^®^ in the USA) which are classified as typical SPIONs, and they are coated with insoluble compounds such as polystyrene for ferristene and siloxane for ferumoxsil. Ferumoxsil is currently the only FDA-approved iron oxide nanoparticle for imaging purposes, specifically for gastrointestinal and bowel imaging [130, 131]. However, the focus is still on IONPs because the scientific community generally agrees that they are safer to use than alternative MRI contrast agents, including Gd-based or Mn-based nanomaterials. Most studies have shown that IONPs are biocompatible in both *in vitro* and *in vivo* testing. Also, contrast agents should retain their colloidal stability since the unfavorable nanoparticle aggregation would cause the T2 and T1 MRI signals to be falsely amplified. Additionally, small molecule-based MRI contrast agents are typically quickly removed from the body by the kidneys following intravenous administration. In contrast, nanoparticle-based agents, especially IONPs, are usually identified and phagocytized by the reticuloendothelial system (RES). Both common pharmacokinetic characteristics shorten blood circulation time, which can negatively impact the quality of contrast MRI. Considering these challenges, research is focused on employing various surface modification techniques to identify small changes in the illness microenvironment that can enhance sensitive MRI activation in the disease defects while longer blood circulation time and retaining colloidal stability.

### Concluding remarks

MRI technology has been revolutionized by the introduction of contrast agents, particularly in molecular imaging. Enhancements in contrast agent stability, relaxivity, and other properties make MRI an effective diagnostic tool for soft tissue disorders. However, recent efforts have focused on developing novel strategies to solve the increasing concern about the safety of Gd- and Mn-based MRI contrast agents, due to the toxicity and potential negative long-term effects accumulation in the body and brain. Regarding this, the FDA approved the first IONP products for clinical applications. Nowadays, IONPs are used in various imaging applications, with ferumoxsil being the only FDA-approved nanoparticle for gastrointestinal imaging. Although major improvement has been made in the field for *in vivo* applications, one major issue is still the possibility that they could cause toxicity when interacting with biological systems and amplifying MRI signals in the disease microenvironment mistakenly. However, even with their increasing use and acceptability, it is still necessary to assess the toxicity of newly created magnetic nanomaterials.

### Acknowledgments

This study was financially supported by Texas Tech University through HEF New Faculty Startup, NRUF Start Up, and Core Research Support Fund. B. R. acknowledges the Distinguished Graduate Student Assistantships (DGSA) offered by Texas Tech University.

The authors wish to thank Mr. Ebrahim Azizi and Dr Rui He for their valuable contributions and input to this contribution of the roadmap.

## 8. MNP-based medical imaging: magnetic particle imaging (MPI)

### Zhi Wei Tay^1^, Chinmoy Saayujya^2^, Quincy Huynh^2^, Jacob Bryan^3^, Renesmee Kuo^3^, Elaine Yu^3^, Prashant Chandrasekharan^3^, Benjamin Fellows^4^ and Steven Conolly^2,3^

^1^ National Institute of Advanced Industrial Science and Technology (AIST), Health and Medical Research Institute, Tsukuba, Ibaraki 305-8564, Japan

^2^ Department of Electrical Engineering and Computer Sciences, University of California Berkeley, Berkeley, CA, United States of America

^3^ Department of Bioengineering, University of California Berkeley, Berkeley, CA, United States of America

^4^ Magnetic Insight, Alameda, CA, United States of America

### Status

**Figure 12.**
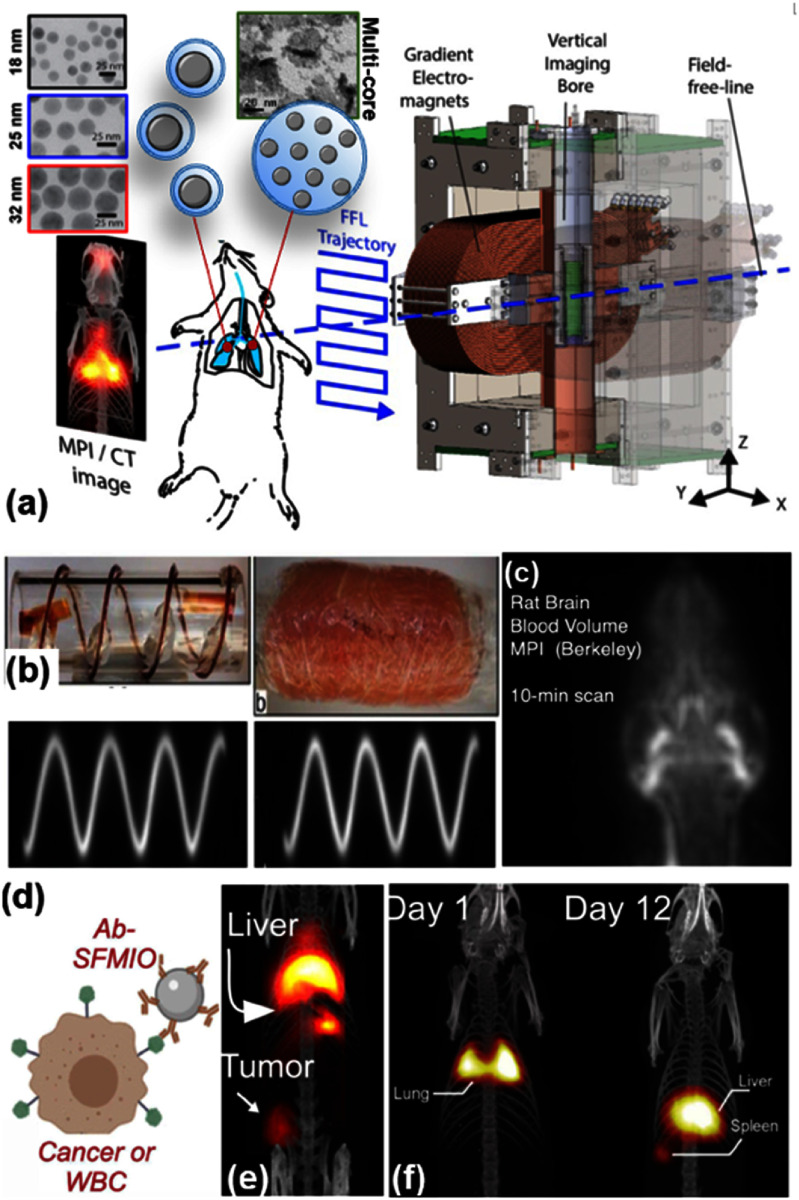
MPI is a new modality that directly & quantitatively images MNPs *in vivo*. (a) MPI scanning schematic where single-core or multi-core MNPs can be imaged *in vivo* even in regions such as the lung where MRI or Ultrasound cannot reach. (b) tissue is fully transparent to MPI, thus (c) enabling direct quantitative visualization of perfusion with zero tissue attenuation. (d) Antibodies can be attached to MNPs enabling specific labeling of cancer cells or stem/immune cells. (e) MPI visualizes tumors with high contrast using EPR (or Ab) targeting. (f) MPI tracks the biodistribution of infused stem cells for longitudinal studies lasting up to 89 d. (a) Reproduced with permission from [134]. [insert copyright line, if specified]. (b) Reproduced from [133], Copyright (2012), with permission from Elsevier. (c) Reproduced from [141]. CC BY 4.0. (d) originally prepared by the authors. (e) Reproduced with permission from [142]. Copyright (2017) American Chemical Society. (f) Reproduced from [143]. CC BY 4.0.

First developed in 2005 by Gleich and Weizenecker [132], MPI is a new imaging technique with distinct hardware and physics from MRI. ^1^H MRI obtains its signal from the nuclear paramagnetism of ^1^H while MPI uses low frequency (kHz) magnetic fields to directly detect the electronic superparamagnetism of iron oxide nanoparticles. While ^1^H has high *in vivo* water concentrations at 55 M and MNPs in MRI generate contrast by affecting this ^1^H signal, Saritas *et al* reported that the electronic superparamagnet-ism detected by MPI is 22 million fold stronger than the nuclear paramagnetism of ^1^H at 7 Tesla, enabling MPI to directly detect nanograms of MNPs [133]. Since there are virtually no superparamagnetic signal sources native to the human body, MPI generates high-contrast tracer-like images of the magnetic nanoparticle (MNP) distribution with zero tissue background [133, 134]. MPI, like PET-CT and PET-MR, requires another anatomic imaging modality to provide anatomic context. Such functional ‘tracer’ modalities that detect only signal emanating from an added reporter/label are fluorescent imaging (FLI), bioluminescent imaging (BLI), and nuclear medicine techniques such as positron emission tomography (PET) and single photon emission computed tomography (SPECT). However, FLI and BLI are limited to around the skin surface due to light scatter and attenuation. Nuclear medicine while capable of deep tissue imaging has limitations of (1) radiation dose (10–30 mSv), (2) cost ($1500–6000/s can due to on-site cyclotron and nuclear biochemist), and (3) trade-offs in dose or sensitivity when using long half-life tracers, and (4) more than 1-hour wait time prior to every study due to the time required for ‘hot chemistry’ binding of the reporter (like Tc99m) to the targeting agent (like Macroaggregated Albumin, MAA), which is a serious challenge for emergency studies, like Pulmonary Embolism (often revealed by MAA-Tc99m Ventilation-Perfusion studies), or Gastrointestinal Bleeds (often revealed by Tc99m-RBC scintigraphy). With further development, MPI has the potential to provide a non-radioactive, deep-tissue ‘magnetic tracer’ imaging complement to existing modalities. Besides MPI, MNPs have also been used as contrast agents in MRI and newer modalities such as microwave imaging for cost-effective breast cancer detection [135] or to modulate hyperthermia of deep-seated tumors [136]. However, the working principle here relies on contrast generation within an anatomic image which differs fundamentally from the magnetic ‘tracer’ principles that is the focus of this chapter. In summary below, MPI has many native advantages as a result of its basis in low-frequency magnetic waves and spatial encoding using the non-linear magnetization of SPIOs [137]:

• **High sensitivity:** MPI harnesses the much higher electronic superparamagnetic signal from SPIOs to improve sensitivity with recent hardware advances toward picogram sensitivity [138].
• **Safety:** MPI uses clinically approved, biocompatible iron oxide agents that emit no radiation dose [137] (figure 12(a)).
• **No view limitations, like CT:** MPI has better penetration into deep tissue than CT with no tissue attenuation or attenuation correction needed as shown in figures 12(b) and (c). MPI works robustly inside the lungs where ultrasound and MRI have limitations.
• **Long persistence *in vivo:*** MPI tracers retain their magnetism until the particle core is digested biochemically (Néelian particles with thick coatings are resistant to aggregation-induced changes in signal). *In vivo* studies show the signal has negligible decay even at 3+ months *in vivo* [139]. In contrast, nuclear medicine tracers have short half-lives of 110 min (FDG), 6 h (Tc99m), or 2.8 d (In111) and require a challenging trade-off between SNR and radiation dose when choosing longer half-life isotopes. MPI poses no such trade-off, see figures 12(e) and (f)).
• **Rapid preparation for emergencies and affordability:** Unlike radioisotopes that must be freshly generated and then bound to the targeting agent just before administration due to rapid signal decay during storage/transport, MPI tracers can be bound in the factory to targeting agents (e.g. MAA, RBCs, WBCs, or antibodies) (figure 12(d)) with much longer shelf life. These imaging agents can be quickly deployed with almost no preparatory time, which will be crucial for rapid emergency diagnoses (pulmonary embolisms, strokes, GI bleeds) [140].

### Current and future challenges

MPI has seen rapid growth in the last decade with the first human-sized head MPI scanners being introduced in recent years. MPI is the only non-radioactive deep-tissue ‘reporter/tracer’ imaging technique. MPI today has excellent safety, contrast, sensitivity, and robustness. With its long persistence *in vivo* and quantitative character, MPI has shown exceptional promise for the longitudinal monitoring and evaluation of stem cell therapies (regenerative medicine) as well as CAR T-cell/adoptive cell transfer immunotherapies for cancer. In addition, MPI is the only non-radioactive deep-tissue modality to robustly image the lung and gastrointestinal tract, with demonstrated success in lung aerosol, lung perfusion, and acute gastrointestinal bleeding. Finally, with its high temporal resolution, MPI has shown promise in dynamic scans of blood volume (with applications in stroke, and functional neuroscience imaging, etc) [144].

One of the most common tracers used in MPI is Ferucarbotran (Vivotrax^TM^). This has worked well in blood pool applications or where cell labeling can be performed *ex vivo*. An exciting ongoing development to improve *in vivo* specificity to biological targets can be addressed partly with antibody-functionalization of the MNP surface. However, compared to the small molecule FDG reporters used in Ab-PET, 10–100 nm MNPs have limited diffusion and extravasation. Unfortunately, the crucial superparamagnetic properties of MPI imaging require a minimum MNP core size [143]. Hence, a key challenge is to optimize MNP synthesis to hit the superparamagnetic ‘core size threshold’ while keeping the overall hydrodynamic size low through very thin biocompatible/stealth coatings that also ensure a long circulation half-life.

MPI’s other major challenge is its spatial resolution at 1.5 mm in small animal scanners. Which is 1 order of magnitude worse than CT, MRI, and ultrasound, which routinely achieve 100-micron resolution. MPI resolution is determined by the applied field threshold where the ensemble magnetization of the SPIO imaging agent starts to magnetically saturate, suppressing signals from off-target-voxel SPIOs [145]. The second factor is the scanner magnetic gradient strength (Tesla/meter) which converts this field threshold into a spatial ‘spot size’. Preclinically, up to a 6.3 T m^−1^ gradient has been achieved. Therefore, reducing the MNP saturation threshold is a critical goal for MPI.

Finally, with regards to the path to clinical translation, iron oxide imaging agents have already been approved for clinical use (i.e. Resovist, Japan) and MPI does not require a strong B0 (7 Tesla) magnet like MRI. However, scaling up preclinical MPI scanners to whole-body clinical size remains challenging due to the reliance of MPI’s spatial resolution on magnetic gradient strength. Cost scales quadratically with gradient strength and the correspondingly larger intra-magnet forces need to be accommodated. As such, many MPI groups have focused on specific body-part imaging such as head scanners, leg scanners or handheld detectors [146–148].

### Advances in science and technology to meet challenges

#### Strategies for improving MPI’s targeting specificity

Targeted delivery of MNPs to specific biological targets often requires (1) avoiding clearance by the RES system (liver, spleen, etc.) and (2) extravasating from the blood circulation to target a pathology, like a tumor. PEGylation is widely used to extend the circulation half-life of MNPs, the liver RES has orders-of-magnitude larger surface area than the target and will absorb most of the circulating MNPs, generating a bright signal in the liver that can overshadow the weaker MPI signal of real targets nearby. One solution is to pre-saturate the liver RES with ‘easily digestible’ non-magnetic nanoparticles preventing the liver MPI signal and increasing MNP accessibility to the target. To address (2), active strategies that employ diapedesis rather than extravasation where the MNP is carried to the (tumor) target by tumor-associated immune cells could be more effective than passive extravasation of the MNPs via the enhanced permeability and retention (EPR) effect, see figure 12(e). Several studies have demonstrated successful *ex vivo and in situ* labeling of such cells [149].

#### Superferromagnetic (SFMIO)-MPI with >10-fold resolution improvement for 100-micron imaging

**Figure 13.**
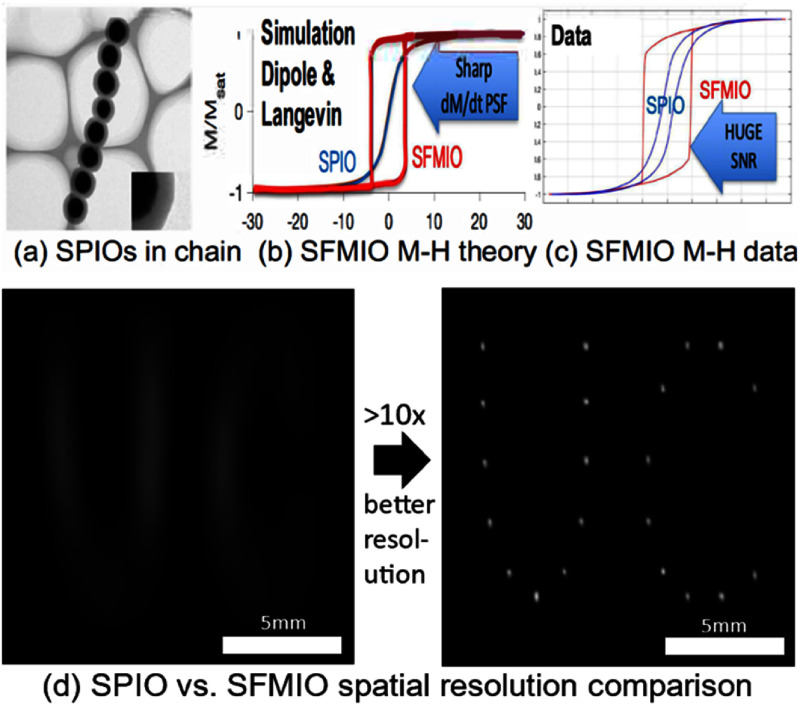
Superferromagnetic iron oxide nanoparticles (SFMIO) enable >10-fold resolution improvement towards achieving 0.1 mm spatial resolution MPI without a limit-of-detection trade-off (SFMIOs also have >10-fold improved SNR concomitant with the improved resolution). (a) TEM shows SPIOs form chains with strong interparticle interactions when magnetized in a field. (b)/(c) SFMIO theoretical simulations match our ‘chained SPIO’ physics model; (d) 2D SFMIOs show ∼30-fold SNR & resolution boost experimentally *in vitro*. (a)–(c) are original figures prepared by the authors. (d) Reproduced from [151]. CC BY 4.0.

The MPI signal detection process relies on broadband inductive sensing. Both SNR and spatial resolution improve proportionately by using MNPs that possess steeper dM/dH slopes for their M(H) saturation curves. The superparamagnetic iron oxide (SPIO) nanoparticles used in MPI obey Langevin physics and saturate at roughly ±5 mT. This saturation value in a 6.3 T m^−1^ gradient translates to a relatively blurry spatial resolution of (10 mT)/6.30 T/m = 1.6 mm. Sharpening the magnetic resolution of SPIOs has been an active research area for more than a decade, obtaining a 2–3 fold resolution improvement. Synthesizing an MPI tracer with a saturation field below 2.0 mT could enable clinical MPI for the first time. Recently, superferromagnetism (SFM) has shown potential to address this challenge. With SFMIO tracers, chains of SPIOs act in concert, displaying hysteresis quite unlike Langevin physics, see figures 13(a)–(c) [150]. SFMIOs have demonstrated switching as fine as 0.4 mT, potentially enabling 100-micron spatial resolutions in MPI. However, this has only worked to date in oil dispersions. New MPI data acquisition and image reconstruction algorithms were tailored for these SFMIOs, to accommodate their coercivity and remanence while realizing order-of-magnitude improvements in both MPI resolution and SNR, see figure 13(d) [151].

### Concluding remarks

MPI is a new biomedical imaging modality centered around the magnetic signal of MNPs to harness them as sensitive non-radioactive ‘tracers’ for a wide range of biological targets. It uniquely leverages the electronic superparamagnetism of the MNPs which is orders of magnitude stronger than the ^1^H nuclear paramagnetic signal in MRI [133], enabling quantitative, sensitive, and direct detection of MNPs *in vivo*. While it is still a nascent field, MPI has seen rapid growth in the last decade with the first human-sized head MPI scanners being introduced in recent years. As a unique non-radioactive deep-tissue ‘magnetic reporter/tracer’ imaging technique, the further development of MPI in combination with high-specificity MNP labels is anticipated to advance the repertoire of imaging tools for clinical molecular biology studies.

### Acknowledgments

The authors gratefully acknowledge support from NIH grants R01s EB029822 and EB024578, U01 EB034694, T32 GM098218, R44 EB029877, M. Cook Chair, Bakar Fellowship, and NSF GRFP.

## 9. MNPs as self-regulated hyperthermia agents

### Ravi L Hadimani^1,2,3^ and Ahmed A El-Gendy^4^

^1^ Department of Mechanical and Nuclear Engineering, Virginia Commonwealth University, Richmond, VA, United States of America

^2^ Department of Biomedical Engineering, Virginia Commonwealth University, Richmond, VA, United States of America

^3^ Department of Psychiatry, Harvard Medical School, Harvard University, Boston, MA, United States of America

^4^ Department of Physics, University of Texas at El Paso, El Paso, TX, United States of America

### Status

Magnetic hyperthermia has been well studied and reported extensively for its potential applications in cancer treatment. Among the many challenges, one of the main challenges in tumor-specific thermal treatment is the spread of heat generated by the alternating magnetic field (AMF) to the surrounding areas thus damaging nearby healthy tissues. It is not possible to maintain temperature uniformity through the tumor during magnetic hyperthermia treatment because of the nonuniform distribution of the applied magnetic field. The tissue closer magnetic field-generating coil will heat up to a higher temperature than the tissue farther away from the coil. The decay of the magnetic field from a dipole source is a function of a cube of radial distance from the source. One of the ways to obtain uniform heating throughout the tissue of target treatment is by using self-regulating magnetic hyperthermia nanoparticles.

**Figure 14.**
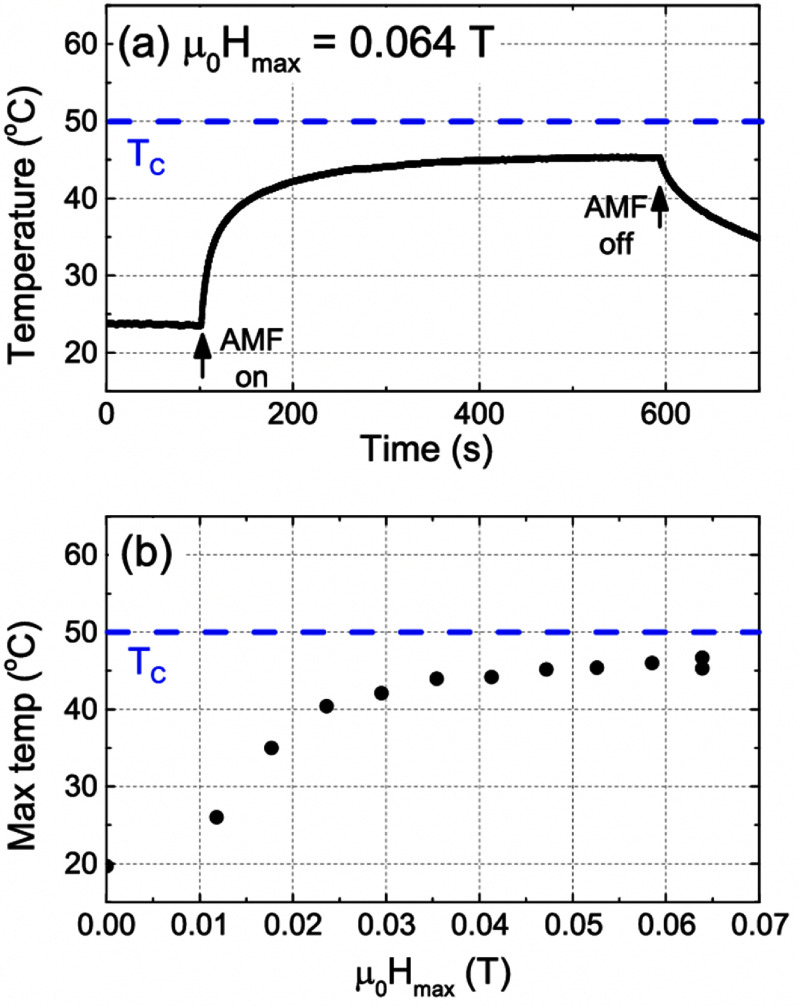
Heating curves and specific loss power (SLP). (a) Heating curves of 0.0326 g of particles with PEG in 500 *μ*l of H_2_O in a flat-bottomed container, were used for SLP calculations. Each period of temperature increase corresponds to a different AMF amplitude. The frequency ranges from 213–217 kHz depending on AMF amplitude. (b) SLP calculated from the slope *dT/dt* in the temperature range 22.1–22.9. © [2017] IEEE. Reprinted, with permission, from [152].

Self-regulating magnetic hyperthermia is a variation of hyperthermia in which the magnetic particles have a magnetic transition temperature *T_C_* around the desired treatment temperature so that magnetic heating only occurs up to that limiting temperature as shown in figure 14 [152]. This can prevent overheating and subsequent damage to healthy cells. It can also help maintain a uniform temperature within the whole tumor volume and at the margins, which otherwise must be achieved by carefully controlling particle distribution and magnetic field strength in the cancerous region, a technical challenge [153]. Several candidate materials have been proposed for self-regulated hyperthermia, typically compounds or alloys in which the stoichiometry can be varied to tune the Curie temperature [152]. One candidate material for self-regulated hyperthermia is Gd_5_Si_4_ and related alloys [154, 155].

### Current and future challenges

Synthesizing mono-dispersed self-regulating magnetic hyperthermia nanoparticles is extremely challenging. Biocompatibility, targeting and delivery, stability, manufacturing scalability, risk-to-therapeutic ratio, and regulatory approval are a few of the challenges in the clinical translation of these nanoparticles. Another main constraint in developing several of these materials is the availability of magnetic materials that undergo magnetic phase transition close to body temperature. Most of the transition metal alloys have magnetic transition temperature/Curie temperature significantly higher than the body temperature. The existing dispersed self-regulating magnetic hyperthermia nanoparticles usually have rare-earth materials such as Gadolinium or Terbium whose transition temperature is close to body temperature and has a high magnetic moment per atom. Bottom-up processes involving hydrothermal processes or other chemical synthesis routes usually involve the reduction or oxidation of rare-earth materials due to their high oxygen affinity. Hence, it is challenging to make dispersed self-regulating magnetic hyperthermia nanoparticles using a bottom-up process. Bulk and thin films of Gd_5_Si_4_ have a high magnetization with *T*_C_= 63 °C, only 20 °C above the desired treatment temperature for hyperthermia [156–158] and it is tunable. Although full cytotoxicity studies have not yet been done, Gd_5_Si_4_ particles are potentially biocompatible. Free Gd^3+^ ions are toxic, but chelated Gd salts are biocompatible and Food and Drug Administration-approved for use as MRI contrast agents [159]. In addition, coated Gd_2_O_3_ nanoparticles are nontoxic and are also candidates for contrast agents [160]. Silicon is biocompatible and Si and Si-based materials are used as coatings for biomedical implants [161]. The high stability of Gd_5_Si_4_ is promising for biocompatibility. The combination of high magnetization, the potential for biocompatibility, and the potential for self-regulated hyperthermia makes these particles an exciting direction for exploration.

### Advances in science and technology to meet challenges

**Figure 15.**
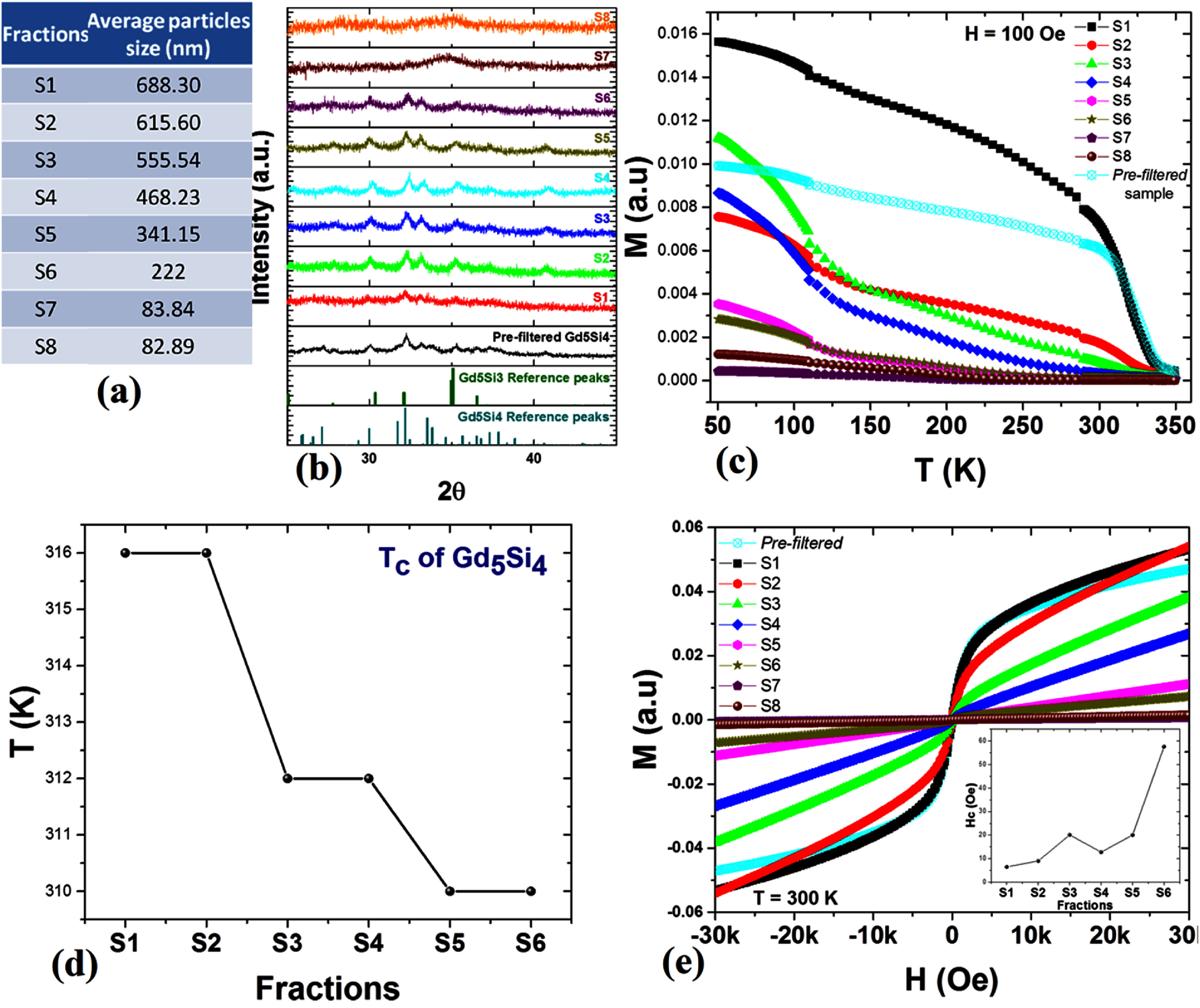
(a) XRD patterns obtained from fractions. Reference peaks of Gd_5_Si_4_ and Gd_5_Si_3_ (bottom) match with the patterns. (b) Gadolinium silicide particle sizes obtained from Scanning electron microscopy (SEM) images of size selected gadolinium silicide particles showing the decrease in average particle size with sedimentation time. (c) M-T curve for all fractions and pre-filtered sample (d) Curie temperatures (*T*_c_) for each fraction (S1-S6) Gd_5_Si_4_ powder. (e) M-H curve for all fractions and pre-filtered sample; the figure inset shows coercivity (Hc) to fractions. Reproduced from [164]. CC BY 4.0.

Top-down processes such as high-energy ball milling or laser ablation have the potential to overcome the challenges described in the previous section. They have two major advantages; mitigation of oxidation of rare earths by working in an inert atmosphere, and the production process is highly scalable. Furthermore, controlling physical properties such as nanoparticle size morphology, composition, etc. can influence the particles’ biological and pharmacological properties, and therefore their clinical applications. These physical properties also influence the magnetic behavior of individual nanoparticles due to size and shape effects [162]. By careful control of process parameters, the morphology and magnetic properties can be controlled as shown in figure 15 [163]. However, the process optimization may involve large parameter space and can be time-consuming. There is a critical need for the development of a scalable process where the oxidation of constituent elements is minimal, and the morphology and magnetic properties can be easily tunable using the process parameters.

### Concluding remarks

Self-regulation magnetic hyperthermia nanoparticles are a potential solution to existing challenges in magnetic hyperthermia such as overheating of tissues and non-uniform hearing of tumours. These nanoparticles overcome these challenges by transforming to a paramagnetic phase above their Curie temperature. The Curie temperature of these nanoparticles is matched to the hyperthermia treatment temperature. These particles can also be prepared by scalable top-down processes such as high-energy ball milling or laser ablation and the transition temperature can be tuned by varying the size of particles.

### Acknowledgments

Authors acknowledge NSF funding for this work: # 1726617, # 1610967, and # 1357565.

## 10. MNPs for neuromodulation

### Renata Saha and Jian-Ping Wang

Department of Electrical and Computer Engineering, University of Minnesota, Minneapolis, MN, United States of America

### Status

Neuromodulation is a rapidly progressing field that has its limitations. Most of the neuromodulation techniques involve the implantation of permanent implants through invasive procedures. In addition, most of these techniques are not equipped to offer spatially selective neuromodulation opening the scope of investigation into other possible techniques. In this section, we discuss the potential of magnetic nanoparticles (MNPs) as neuromodulators. When exposed to an alternating magnetic field (AMF), these MNPs radiate heat, mechanical force/torque, and/or electric field at the nanoscale through hysteresis. For the treatment of cancer, these MNPs have already shown extensive promise due to the fact they can be delivered remotely, non-invasively, and wirelessly. It is because of these exciting properties, that MNPs are being experimentally studied for their usage in the treatment of neuropsychiatric disorders.

**Figure 16.**
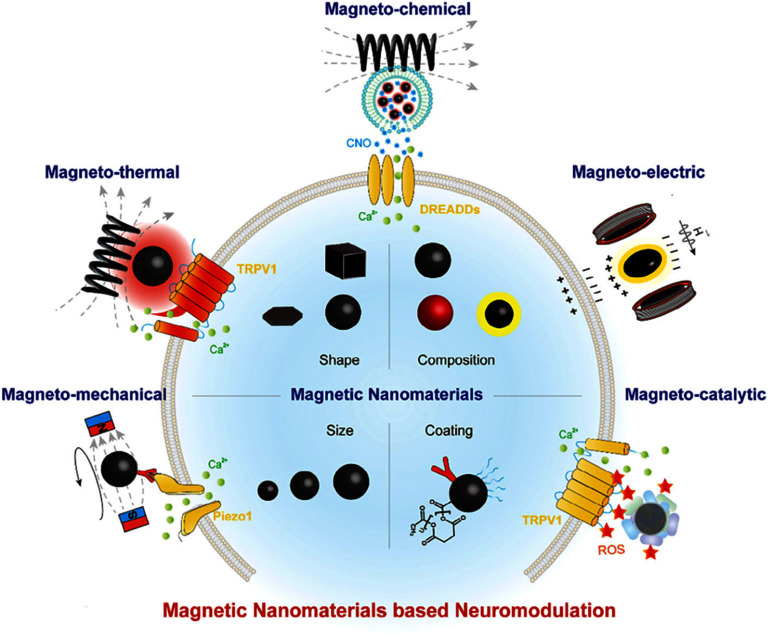
MNP-mediated neuromodulation demonstrates the activation of cellular ion channels through various techniques, including magneto-mechanical, magneto-thermal, magneto-chemical, magneto-electric, and magneto-catalytic approaches. External magnetic fields can activate MNPs. Specifically, magneto-mechanical forces can activate Piezo1, a mechanosensitive ion channel. The potential for magnetic drug delivery arises, wherein designer G-protein-coupled receptors like DREADDs can be activated under an AMF. Additionally, the magneto-electric effects of MNPs can induce neuronal depolarization under radiofrequency conditions, while the magneto-catalytic effects of MNPs can activate TRPV1 coupled with ferritin [172]. John Wiley & Sons. [© 2023 Wiley Periodicals LLC.].

Neurons are genetically engineered to express specific ion channels (such as TRPV1, TMEM16A, TRPV4, and PIEZO1) to trigger neural activation. These specific ion channels respond to external stimuli mediated by MNPs, including mechanical force/torque (magnetomechanical stimulation [165]), heat (magnetothermal stimulation [166]), electricity (magnetoelectric stimulation [167]), and chemical substances (magnetochemical stimulation [168]). During stimulation, these ion channels open, allowing ions to enter neuronal cells to excite or inhibit them (see figure 16). MNP-mediated neuromodulation provides a means of accessing deep brain regions with high specificity through a remote, non-invasive, and untethered approach, distinguishing it from other neuromodulation techniques [169]. Over the last decade, significant strides have been taken to enhance MNP-mediated neuromodulation methods. These efforts include fine-tuning the magnetic properties of MNPs, engineering ion channels, incorporating chemical stimuli, and developing transgene-free techniques. Consequently, MNP-mediated neuromodulation has yielded insights into the functional connectivity of neural circuits, motor behaviors, and potential treatments for neuropsychiatric disorders [166, 170, 171]. Despite these advancements, MNP-mediated neuromodulation is still in its early stages and requires further refinement for more advanced applications in neuroscience.

### Current and future challenges

Despite numerous research efforts showcasing advancements in the field, the technique of using MNPs for blood-brain barrier (BBB) crossing and drug delivery remains confined to the proof-of-concept stage. Existing reports are restricted to rodent models, with no exploration of non-human primates. To date, there has been no genuine commercial success in applying MNPs for *in vivo* biomedical purposes. Their use has been limited to serving as enhanced contrast agents in nuclear magnetic resonance (NMR), exemplified by Feridex in the US, Berlex Laboratories; Endorem in the EU; Resovist in the EU and Japan; and Cliavist in the EU, among others. One significant challenge hindering the clinical transition of this technique is the rapid decrease in magnetic field strength with distance, particularly complicating the control of MNPs using an external magnetic field on obese patients. The creation of a magnetic trap using a static magnetic field is nearly impossible, necessitating the achievement of magnetic focusing through dynamic fields and their corresponding space-time averages. Furthermore, during the propulsion of MNPs to the target location, careful consideration must be given to prevent the applied external AMF from dispersing other essential particles at the target location. Additionally, MNPs exhibit an extremely poor retention rate of the magnetic field. Consequently, once the external AMF is discontinued, the MNPs struggle to remain near the target location and may disperse across the region, complicating the consolidation of all MNPs in subsequent AMF cycles. An additional concern involves the exit path for these MNPs from the system following neurostimulation technology. Questions arise about the fate of injected MNPs—where do they go within the body? What metabolic processes do they undergo? Are there potential issues related to neurotoxicity? These are critical inquiries that researchers in MNP-mediated neurostimulation must earnestly address.

### Advances in science and technology to meet challenges

MNP-mediated neuromodulation achieves non-destructive activation of ion channels. However, the invasive nature of stereotactic MNP injection to reach the target region remains a challenge. Consequently, it is imperative to conduct a study on the dynamics of MNPs within the brain microvasculature system. In a study by Kosari and Vafai [173], the authors highlight the possibility of thermally stimulating brain blood vessels without the need to breach the BBB or employ invasive nanoparticle delivery methods. They introduce a novel neuromodulation technique that involves exciting MNPs safely delivered to the microvasculature in proximity to the target tissue. The approach leverages the transport and dynamic interactions within the brain capillaries as blood flow carries the heat generated by the MNPs.

### Concluding remarks

The distinctive characteristics of MNPs render them ideal for targeting the central nervous system (CNS) to optimize various biomedical applications such as drug delivery, neuromodulation, and magnetic hyperthermia. Typically, superparamagnetic nanoparticles are employed for these purposes, and their inherent property sets an upper limit on their size, thereby restricting the potential size of MNPs that can breach the BBB. In this section, we discussed existing MNP-mediated neuromodulation techniques, including magnetothermal, magnetoelectric, chemomagnetic, and magnetogenetic methods. MNPs utilized in neuromodulation efficiently convert magnetic fields into heat, mechanical force, and electrical energy, showcasing extensive catalytic capabilities. We delved into how MNPs provide targeted, spatially selective, non-invasive, and wireless neuromodulation by influencing specific ion channels on the neuron membrane. Lastly, we addressed some of the challenges hindering the progression of MNP-mediated neuromodulation into clinical trials. A comprehensive examination of the safety aspects of MNPs, encompassing potential thermal damage to tissues, stereotactic injection into the system, and neurotoxicity, is crucial. The development of technologies facilitating targeted and focused neuromodulation, particularly in primates, represents an urgent mission, and MNP-mediated neuromodulation is steadily advancing in that direction.

### Acknowledgments

R S acknowledges the 3 yr College of Science and Engineering (CSE) Fellowship awarded by the University of Minnesota. R S was supported by the University of Minnesota’s MnDRIVE (Minnesota’s Discovery, Research and Innovation Economy) initiative. J-P W acknowledges the financial support from the Institute of Engineering in Medicine and the Robert F. Hartmann Endowed Chair professorship by the University of Minnesota.

## 11. Magnetic nanoparticles for tissue engineering

### Thomas J Broomhall^1^, Abigail L Wright^1^, Michael Rotherham^1,2^ and Alicia J El Haj^1,2^

^1^ Healthcare Technologies Institute, School of Chemical Engineering, University of Birmingham, Edgbaston, Birmingham, United Kingdom

^2^ National Institute for Health and Care Research (NIHR) Birmingham Biomedical Research Centre, Institute of Translational Medicine, Birmingham, United Kingdom

### Status

#### Introduction to tissue engineering with magnetic nanoparticles

Tissue engineering (TE) aims to replace diseased or damaged tissue that cannot be treated using conventional therapies. TE uses key components of the tissue environment such as growth factors, stem cells, and a mechanically tailored scaffold material to build tissues *ex vivo*. Recent advances have recognized the importance of engineering the stem cell niche and identified the importance of tissue mechanics and topographic cues for ordering cells within their engineered tissues. Once constructed, the aim is to implant the TE into the patient to promote host integration, healing, and structural support. Besides these components, an array of enabling technologies are required to monitor, augment, and fine-tune tissue-engineered constructs to produce functional tissue [174]. In recent years magnetic nanoparticles (MNP) have become a key part of the TE toolkit. The combination of MNP and TE often focuses on precise tracking, imaging, and control of cells and the cellular environment, either through mechanical actuation, spatial control, or the development of scaffolding systems. By leveraging the variable biofunctionalization of MNP coatings, a range of cellular or biomaterial targets can be employed. The variety of targeting options, and control mechanisms can enable control of cell adhesion, proliferation, and differentiation. Thereby rapidly increasing the growth and repair of cell constructs.

#### MNP for biomedical imaging and targeting

The magnetic properties of MNP provide an accessible and non-invasive way of imaging and tracking MNP-labelled cells or monitoring the integration of engineered tissue constructs using imaging modalities such as MRI. Magnetically tagging cells also enable cell therapies to be targeted, localized, and retained at repair sites by using external magnets to guide the tagged cells to the target site [175, 176].

#### Static magnetic fields and cellular alignment

**Figure 17.**
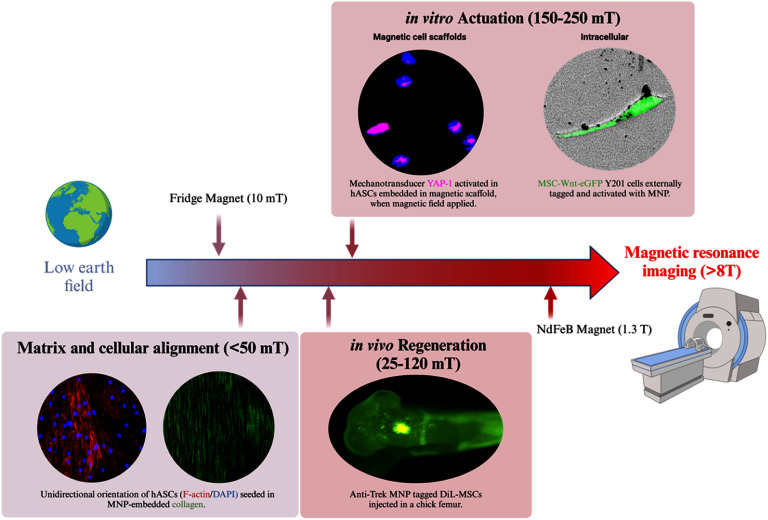
Applications of magnetic fields in biomedical engineering. Low flux density magnetic fields (<50 mT) can align MNP-tagged cells or matrix proteins. Increasing magnetic flux densities (25–250 mT) can initiate magnetic actuation of cells *in vitro* and *in vivo* for regenerative medicine applications. Higher magnetic flux densities (>8 T) are often required for magnetic resonance imaging. Created with BioRender.com.

MNPs, attached to cells, can be used to control the direction of growth and alignment of cells and tissues, to create aligned gels and patterned constructs (figure 17). Due to the strong magnetic response of an MNP in a static magnetic field, the particles are ‘dragged’ along the field direction, creating linear arrays of particles and cells [177]. More complex structures, such as cell sheets as tissue patches and 3D scaffolds can be deployed to enable multiple cell types to be investigated in relevant biomimetic environments [178].

#### Actuation

Magneto-mechanical actuation of cells has applications in several biological niches. By specifying the biofunctionalized coating of the MNP to target a select cellular receptor or ion channel, MNPs can be used to trigger mechanotransduction in a range of cell types by inducing movement of the MNP (figure 17). The movement of MNPs generates small (often on the femto- or pico-Newton range) forces which are transferred to the cell exterior. This triggers mechanically sensitive pathways in a non-invasive manner to remotely control the cell behavior [179].

### Current and future challenges

#### Safety

Within benchtop settings, the biocompatibility of iron-oxide-based MNPs has been extensively studied and is found to be biologically safe [180]. However, to enable confidence in the translation of these MNP-mediated applications there needs to be further studies into long-term effects in patients *in vivo*, or those arising from more extensive usage in future therapies. Further study should be carefully approached, as the variety of factors that can influence safety (i.e. particle size, functionalization, protein corona, concentration) could raise concerns until the safety is better established per particle parameter [181].

#### Field strength

One key limiting factor of scaling up MNP-mediated therapies to the human scale is the penetration depth and strength of magnetic fields. Commonly, the field generated by a simple bar magnet decays at a rate of 1/*r*^3^, often this is measured as exponential decay in lab settings. This raises the challenge of generating and controlling a focused magnetic field of sufficient strength at relevant depths to achieve magnetic stimulation in deep tissue.

#### MNP variability

To ensure consistent MNP response to magnetic fields, it is necessary to maintain consistent MNP parameters such as size, magnetic material and the biocompatible coating concentration. Small variations in MNP diameter, whether a singular particle or an ensemble of particles in one MNP structure can drastically alter the magnetic response of the particle to an externally applied magnetic field. Alongside the size of particle, the material phase is also of importance, especially when using Fe_2_O_3_ nanoparticles due to the differences in saturation magnetization between different phases of iron oxide (magnetite/maghemite for example) [182]. Variation in the concentration of biocompatible ligands may also alter the binding behaviour of the MNPs to cells, arising from loading or size differences in the particles, and will also affect cellular responses. Therefore, it is imperative that steps are taken to ensure rigor and consistency within MNP fabrication, quality assurance steps, and experimental handling with regards to coating and loading of cells with biocompatible ligands.

#### Multiple cell types

Tissues and organs are composed of cells from multiple lineages, which are spatially arranged hierarchically. To repair and regenerate functional tissues, multiple cell types must be spatially arranged at different stages of differentiation, and their differentiation cues should be precisely controlled using variable forces. Previous work has shown the use of MNPs to arrange and align cells *in vitro* and to trigger differentiation pathways in isolated cell populations using magneto-mechanical force [177–179]. However, the major challenge of optimizing these processes still exists. Due to the range and complexity of cellular pathways required for correct tissue patterning, and the variability in MNP response with spatially varying magnetic fields, a clear and thorough understanding of the signalling events controlling cell behaviour for each cell or tissue type is required. It is also essential that the cellular response to magnetic materials is shown to be consistent and controllable when developing MNP-mediated TE therapies. The success of MNP as a regenerative TE tool will ultimately require the tagging of multiple receptor targets, in multiple cell types, using variable forces, and in cells that have been precisely positioned in the correct sequence in 3D.

#### Translation to clinical settings

The ultimate aim for TE therapies is their deployment into clinical settings, however translating from lab-based methods to patient-centred technologies often poses additional challenges. In lab-based trials, each facet of the therapy is at the forefront of its respective field (biomaterials, engineering etc) and the experimental process is readily tailored and highly controlled. Whilst this poses specific challenges in a laboratory, enabling these therapies to be used by healthcare professionals, patients or carers also comes with other non-trivial developmental concerns. Ensuring a therapy, and any associated medical devices, can be easily applied by a range of end-users, whilst also ensuring complex pathways are targeted and stimulated effectively, can introduce variability and make final intervention development vary from lab testing. These issues should be considered throughout all stages of experimental planning to ensure consistency in the safety and efficacy of the final therapy. Further challenges can arise when seeking regulatory accreditation, as different standards are required for medical devices and medicinal products, therefore developing the experimental process and device design with these in mind may help support the translation from lab to clinic.

### Advances in science and technology to meet challenges

#### Halbach arrays

Due to the limitations of magnetic field strengths and shapes over long distances from singular permanent magnets, more complex magnetic arrangements can be used to increase field strengths and create controlled field shapes. One key approach commonly used is the Halbach Array, in which an array of permanent magnets is rotated such that the resulting field becomes highly directional and results in a stronger magnetic field from one side of the array. This can enable field shapes to be tailored towards a target area whilst minimizing stray magnetic fields. By increasing the number of Halbach arrays, and their positionings it is possible to create complex field shapes, including circular field arrangements.

#### 3D patterned arrays

Extending on from simple Halbach arrangements, more complex 3D structures can be employed. These can range from irregularly shaped permanent magnets to more spatially complex arrangements [183, 184]. By optimizing array designs to a given target construct, novel field profiles may be achieved which can target therapies in larger tissue constructs.

#### Electromagnets

Another approach for generating magnetic fields of sufficient strength at a distance is using electromagnets. By employing time-varying currents through coils, it is possible to vary the magnetic field strength mimicking the motion of permanent magnet arrays. More complex arrangements of coils can be used also to create spatially varying magnetic fields [185].

#### Bioprinting

The inclusion of MNPs into hydrogel structures can be used to tailor not only a magnetic response but can also be used to alter the rheological properties of the hydrogel. This not only enables a magnetically induced response in a final structure but can also be used to aid in generating 3D structures and the generation of bio-inks. By including MNPs in the 3D fabrication step, scaffolds can be developed to apply global stresses to cell constructs under applied magnetic fields or to enable more even heating from magnetic-hypothermia treatments.

### Concluding remarks

MNPs have significant promise as enabling tools for TE across a range of applications; from biomedical imaging, cell tracking, and promoting cell alignment to cellular control and mechanotransduction. Alongside improvements in engineering approaches for the development of 3D scaffolds, the incorporation of magnetic components has also demonstrated the value that MNPs provide in creating complex multi-cell tissue structures. This can be achieved through targeted cell control platforms, varying rheological properties of bio-inks, and leveraging global stresses on cell constructs through magnetically responsive scaffolds. Developments in the engineering of more complex magnetic field structures and devices have also furthered the application of MNPs into wider settings, and further work will help drive the translation of benchtop tissue engineering to clinical settings.

### Acknowledgments

We acknowledge financial support from an EU ERC Advanced Grant DYNACEUTICS (Grant No. 789119), and by the National Institute for Health and Care Research (NIHR) Birmingham Biomedical Research Centre (BRC), Grant No.: NIHR203326. The views expressed are those of the author(s) and not necessarily those of the NIHR or the Department of Health and Social Care.

## 12. MNPs for drug and gene delivery

### Zhiyi Wang and Jiarong Liang

Spin-X Institute, School of Chemistry and Chemical Engineering, State Key Laboratory of Luminescent Materials and Devices, South China University of Technology, Guangzhou, Guangdong Province, People’s Republic of China

### Status

Magnetic nanoparticles (MNPs), particularly iron oxide variants like magnetite (Fe_3_O_4_) and maghemite (γ-Fe_2_O_3_), exhibit a key feature known as superparamagnetism. This property allows them to become magnetized in the presence of an external magnetic field and lose their magnetism once the field is removed, preventing aggregation and enhancing biocompatibility. Surface modification is essential for MNPs, influencing their stability, cellular uptake, and immune response.

**Figure 18.**
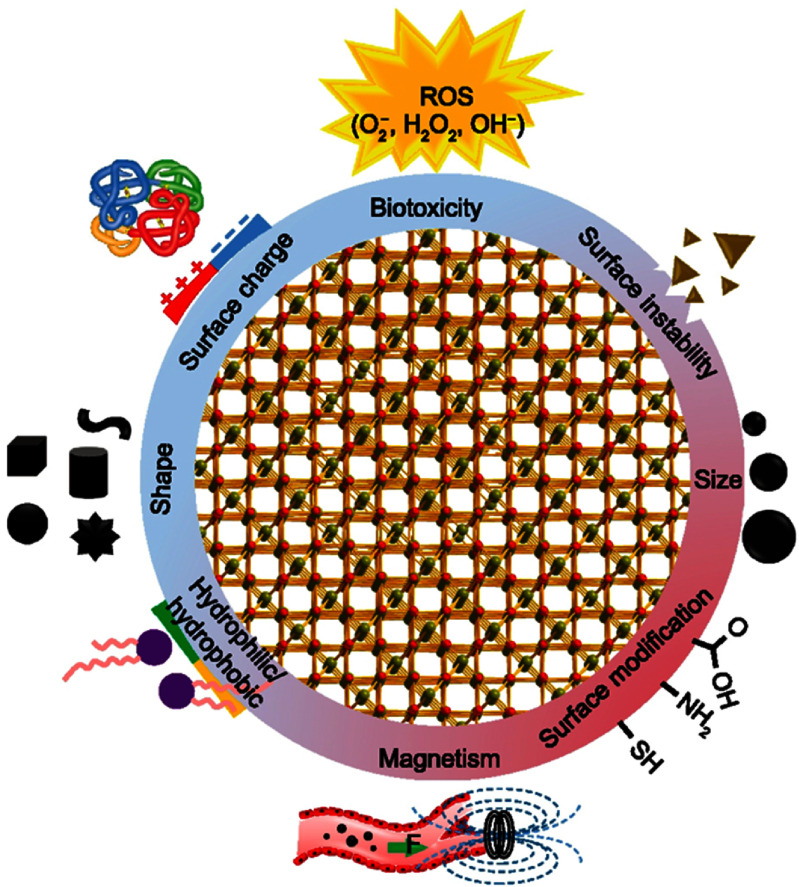
Physicochemical characteristics of MNPs for drug delivery systems. Reproduced from [186], with permission from Springer Nature.

As shown in figure 18, to improve these characteristics, MNP cores are often coated with materials such as polymers (e.g. polyethylene glycol or polyvinyl alcohol), inorganic substances (e.g. silica or gold), or biomolecules (e.g. antibodies or peptides). These coatings stabilize the nanoparticles and provide functional groups for drug conjugation or targeting moieties. Drug loading onto MNPs can be achieved through physical adsorption, where drugs attach via electrostatic or hydrophobic interactions, or through covalent bonding for a more stable attachment [186]. Encapsulation methods, such as embedding drugs within a polymer or liposome shell around the MNP core, offer additional control over drug release.

These design and functionalization strategies enable precise targeting, controlled drug release, and minimized toxicity, making MNPs versatile and efficient tools in advanced drug delivery. MNPs are designed to specifically target affected regions, minimizing the impact on healthy tissues [187]. Their ability to be guided by magnetic fields enhances their value in drug delivery, evolving from mere diagnostic tools to theranostic agents that offer both diagnostic and therapeutic capabilities [188], especially in cancer treatment.

The mechanisms of drug delivery using MNPs leverage their magnetic properties to enhance therapeutic efficacy. MNPs can be directed to specific sites within the body using external magnetic fields, concentrating the therapeutic agents at the target site and minimizing systemic exposure. Once localized, drug release can be controlled by various stimuli, such as pH changes in the tumor microenvironment, temperature variations, or alternating magnetic fields, allowing for precisely timed therapeutic actions. Hyperthermia-induced release is another mechanism, where MNPs generate localized heat upon exposure to an alternating magnetic field, facilitating drug release and providing therapeutic effects through hyperthermia. MNPs can also be engineered for stimuli-responsive release, where the drug is released in response to specific biological signals like enzyme activity or redox conditions [189, 190].

This versatility ensures that MNPs provide sustained and controlled therapeutic effects, enhancing overall treatment outcomes. Additionally, their ability to deliver drugs and act as imaging agents for real-time monitoring highlights their potential in advanced drug delivery systems, making them powerful tools for targeted and controlled therapeutics.

### Current and future challenges

The field of MNPs, particularly superparamagnetic iron oxide nanoparticles (SPIONs), for drug and gene delivery presents several key research issues and challenges. The size of SPIONs is crucial for their effectiveness in drug delivery. They must be small enough (ideally under 200 nm) to evade the immune system yet large enough to carry sufficient therapeutic payload. Achieving the right balance between size, stability, and functionality is a significant challenge.

Additionally, maintaining colloidal stability in biological environments without aggregation is a major research focus [191]. Ensuring biocompatibility and minimizing toxicity of MNPs is paramount. Researchers must continually assess the long-term effects of these nanoparticles in the body, particularly their interaction with the immune system and potential accumulation in organs.

Developing coatings or surface modifications that enhance biocompatibility without compromising functionality is a significant challenge. Achieving high targeting efficiency while minimizing effects on non-targeted tissues is a critical issue. This involves improving the precision of magnetic guidance and enhancing the specificity of MNPs to target cells or tissues. Overcoming biological barriers, such as the blood-brain barrier, to deliver drugs to hard-to-reach areas remains a substantial challenge.

Advancing the development of controlled and on-demand drug release mechanisms at target sites represents a pivotal research domain. This encompasses the creation of responsive systems capable of releasing medications in response to specific triggers such as pH variations, temperature fluctuations, or external magnetic fields. While MNPs have proven effective as contrast agents in MRI, there remains a pressing need to enhance their imaging capabilities, focusing on improving resolution and specificity to more accurately monitor treatment progress.

Meeting the challenge of scaling up MNP production while ensuring consistent quality and functionality is a priority, necessitating the formulation of cost-effective and reproducible manufacturing processes tailored for clinical applications [192].

Translating MNPs from laboratory research to clinical applications faces several significant challenges. Demonstrating the long-term safety and efficacy of MNPs in clinical settings is critical. Long-term studies are needed to monitor potential delayed adverse effects, stability of the nanoparticles, and their sustained therapeutic impact. Addressing concerns such as the potential degradation of coatings, the release of toxic ions, and chronic inflammatory responses is essential for gaining clinical acceptance. The use of MNPs in medicine also raises ethical and social considerations. Issues related to patient consent, potential risks versus benefits, and the equitable distribution of advanced nanoparticle-based therapies need careful consideration. Public perception and acceptance of nanotechnology in medicine play a crucial role in its clinical translation.

### Advances in science and technology to meet challenges

Advances in the science and technology of magnetic nanoparticles are crucial in overcoming existing challenges and harnessing their full potential in drug and gene delivery. MNPs are highly biocompatible and possess a range of properties useful for drug delivery. Their use as contrast agents in MRI, as well as their ability to be guided by magnetic fields and induce local heating in tumor regions for drug release or apoptosis, positions them as advanced theranostics agents [193]. However, the tendency of MNPs to aggregate necessitates combining them with biological or synthetic polymers, which enhances their stability and allows for secondary functionalization with drugs and protective compounds against immune recognition. The intrinsic magnetic properties of MNPs enable strong responses to small magnetic fields. Their superparamagnetic nature emerges when the nanoparticle size falls below a threshold, enhancing imaging contrast and manipulability by magnetic fields. This property is crucial for drug targeting, especially in deep body locations like the brain. MNPs’ biodegradable and biocompatible nature, coupled with efficient clearance from the body, makes them highly suitable for medical applications. Magnetically engineered drug delivery systems (MEDDS) involve combining MNPs with other materials to create hybrid structures with new properties and functionalities. This includes systems where drugs and MNPs are enclosed separately, allowing for selective and conditional release in response to biochemical or physical stimuli. Integrating MNPs with macromolecules to form these complex structures is vital for maintaining stability in biological environments and achieving specific drug delivery functions. To address these challenges, ongoing research must focus on developing new synthetic methods to produce MNPs with controlled size and shape, integrating them with other materials to enhance functionality, and tailoring their surface properties for specific biomedical applications. This will enable more effective and targeted drug delivery, improved diagnostics, and overall better therapeutic outcomes.

### Concluding remarks

In conclusion, the advancement of MNP technology is pivotal for overcoming challenges in drug and gene delivery. Key developments include enhancing biocompatibility and multifunctionality by combining MNPs with biological or synthetic polymers, which not only stabilizes them but also allows for sophisticated functionalization with therapeutic agents and protective compounds. The exploitation of MNPs’ magnetic properties is crucial for targeted drug delivery, especially to deep and hard-to-reach body areas. This requires precise control over their size and magnetic properties. Additionally, the design of MEDDS that integrates MNPs with other materials is essential to create hybrid structures for selective and conditional drug release. Advancing these areas will enable more effective, targeted therapies and diagnostic capabilities, ultimately enhancing the potential of MNPs in medical applications.

### Acknowledgments

This work was financially supported by the National Natural Science Foundation of China (52001008), and the Guangzhou Basic and Applied Basic Research Foundation (202201010669).

## 13. Cellular uptake and degradation of MNPs

### Ana Abad-Díaz-de-Cerio^1^, Lucía Gandarias^2, 3^, Alicia G Gubieda^1^, Ana García-Prieto^4^ and Mª Luisa Fdez-Gubieda^3^

^1^ Dpto. Inmunología, Microbiología y Parasitología, Universidad del País Vasco–UPV/EHU, Leioa, Spain

^2^ Bioscience and Biotechnology Institute of Aix-Marseille (BIAM), Aix-Marseille Université, CNRS, CEA—UMR 7265, Saint-Paul-lez-Durance, France

^3^ Dpto. Electricidad y Electrónica, Universidad del País Vasco—UPV/EHU, Leioa, Spain

^4^ Dpto. Física Aplicada, Universidad del País Vasco–UPV/EHU, Bilbao, Spain

### Status

The biocompatibility, *in vivo* biodistribution, cellular uptake, and fate of magnetic nanoparticles (MNPs) depend on the composition, size, morphology, surface coating, and functionalization of the nanoparticles as well as on the cell type they are in contact with. Understanding the mechanisms of cellular uptake of MNPs is mandatory to optimize the delivery efficacy to the target cells and to avoid their clearance by immune system cells.

**Figure 19.**
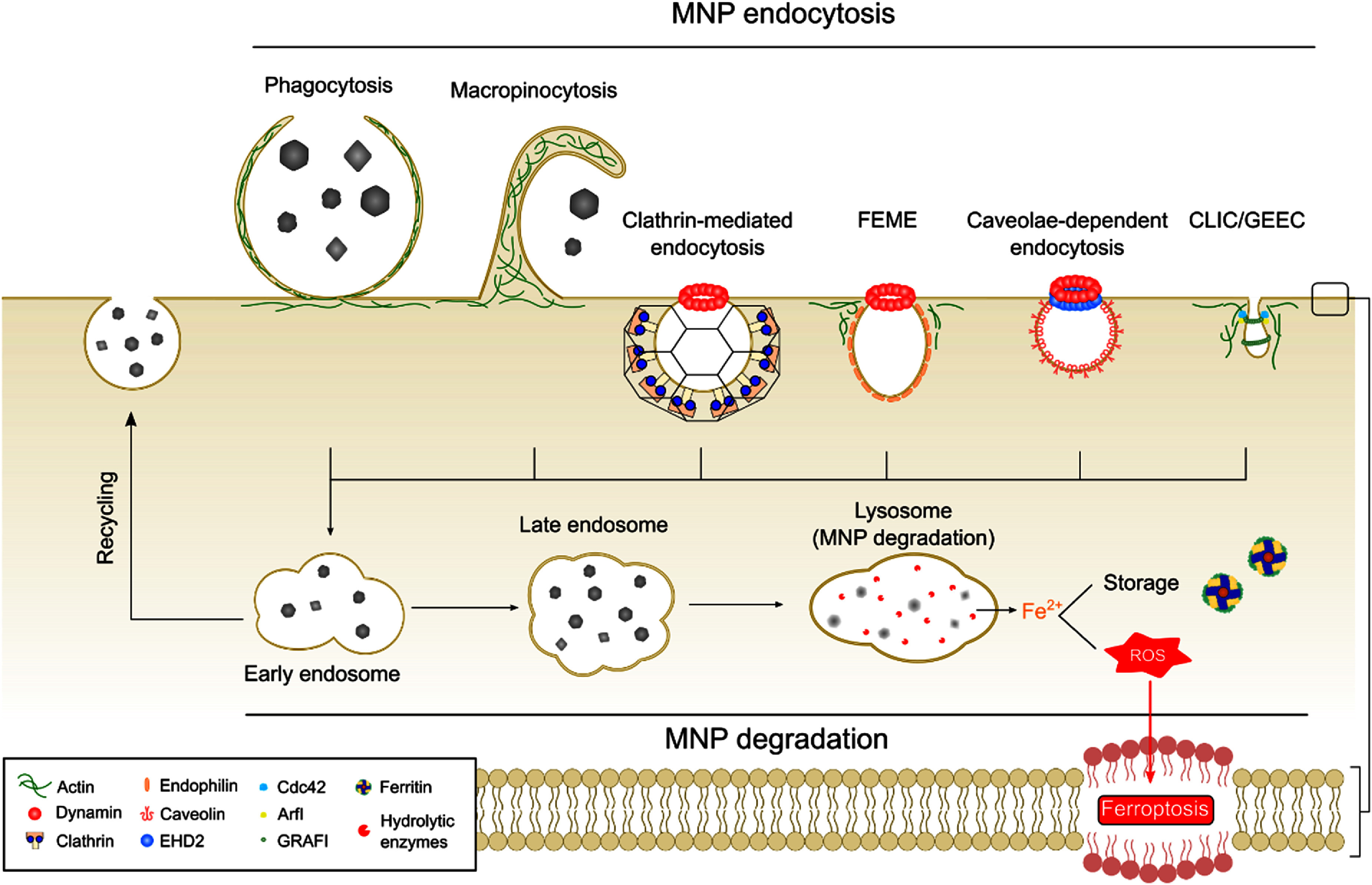
Overview of the possible endocytosis pathways for MNP entry into cells and MNP intracellular fate. FEME (fast endophilin-mediated endocytosis), CLIC/GEEC (clathrin-independent carrier glycosylphosphatidylinositol-anchored protein-enriched early endocytic compartment endocytosis), EHD2 (Eps15-homology domain-containing protein 2), Cdc42 (cell division cycle 42), ArfI (ADP-ribosylation factor 1), GRAFI (GTPase regulator associated with focal adhesion kinase-1), ROS (Reactive Oxygen Species). Original figure prepared by the authors.

MNPs enter cells mainly by endocytosis. At present, there is a consensus for six different endocytic pathways [194] (figure 19). All these pathways have in common that the internalized cargo ends up being stored in early endosomes. Here, the cargo can be either recycled back to the cell surface and expelled or go onwards to late endosomes that fuse with lysosomes for degradation. Tracking the degradation of MNPs inside cells is crucial for assessing their therapeutic potential. To date, most of these studies have been focused on iron oxide MNPs.

Iron oxide nanoparticles are generally considered safe, biocompatible, and non-toxic materials with reported LD-50 (the median lethal dose or the dose required to kill half of the tested animals during a specified time) of 300–600 mg Fe/kg body weight for uncoated MNPs and 2000–6000 mg/Fe kg for MNPs coated with biocompatible dextran molecules [195]. The toxicity of different MNPs has been studied for different cell lines and *in vivo*, generally presenting no apparent abnormal changes or mild toxicity [196–198]. However, depending on dose, coating, and route of administration, MNPs may alter some metabolic pathways, immune responses, or inflammation [197].

*In vitro* studies demonstrate a rapid degradation of maghemite NPs (Fe_2_O_3_) in stem cells within a month [198], and storage of the released iron in ferritin. X-ray absorption near edge structure (XANES) measurements with magnetite NPs (Fe_3_O_4_) in macrophages and lung cancer cells show that after 20 days only 45% of the iron remains as magnetite, 40% as maghemite, and 15% is stored in ferritin as ferrihydrite. The degradation process is faster in macrophages than in cancer cells [199] and in 2D cultures than in spheroids. Interestingly, Van de Walle *et al* [198] report on the ability of stem cells to biosynthesize magnetic nanoparticles anew from the degradation products.

Finally, *in vivo* studies revealed that iron oxide MNPs are mainly retained in the liver and spleen due to the abundance of macrophages in these organs [200–203]. Zelepukin *et al* [203] have shown that smaller MNPs degrade faster, attributed to the higher surface area/volume ratio. At the same injection dose, a higher particle surface area available for degradation leads to an acceleration of the particle destruction rate, being much faster for MNPs with negatively charged coating than for those with positively charged coating. Furthermore, the MNP coating determines the proteins that will be adsorbed into it and form the protein corona, which has also been related to the degradation of the magnetic core [204]. Degradation rates increase with MNP dose, up to a saturation point. Notably, the internal particle nanoarchitecture is crucial, with uniform multicore particles degrading faster than core-shell multicore particles [203].

### Current and future challenges

The successful implementation of MNPs in biomedical applications relies on achieving an efficient cellular uptake. Key challenges that need to be addressed span from a better understanding of the internalization and degradation mechanisms at the cellular level, to the efficient delivery of MNPs to the target cells.

The classical approach to study nanoparticle endocytosis pathways is pharmacological inhibitors, but these inhibitors can cause cytotoxicity and/or non-specifically block more than one endocytosis route. Another challenge to face when working with pharmacological inhibitors is that the techniques commonly used to determine MNP uptake (i.e. flow cytometry or microscopy) often do not allow distinguishing the localization of MNPs in the cells (e.g. inside or attached to the plasma membrane).

Upon entry into the cells, understanding the impact of MNP degradation on their therapeutic and diagnostic potential becomes crucial. The process of degradation presents both advantages and challenges. On the one hand, the degradation of MNPs has the potential to diminish their theranostic capabilities, affecting treatments like magnetic hyperthermia and photothermia [205], and magnetic contrast imaging. On the other hand, the degradation of MNPs can lead to the release of Fe^2+^ ions, a highly toxic compound known to elevate reactive oxygen species (ROS) production within cells, thereby inducing ferroptosis, a form of programmed cell death. This unique attribute positions MNPs as promising candidates for chemodynamic and ferroptosis therapies [198], presenting opportunities for combined applications with magnetic hyperthermia and photothermia. Another current challenge is the lack of knowledge on how the application of treatment could affect the degradation process of MNPs. Recent findings suggest that laser-mediated temperature increases during photothermal treatment can accelerate the degradation of MNPs [206].

Finally, a significant challenge is scaling up from an *in vitro* cell monoculture to a complex environment *in vivo*. This includes, among others, understanding the interactions with other cells and proteins/lipids to avoid recognition by the mononuclear phagocyte system or enhancing MNPs’ capability to overcome biological barriers, a critical aspect for achieving deeper penetration and more homogeneous distribution of MNPs within tissues.

### Advances in science and technology to meet challenges

Recent advances to determine the MNPs’ cellular internalization routes avoiding the unspecificity associated with pharmacological inhibitors point to the use of knockouts of key components of the different endocytosis pathways using siRNA [194]. In addition, instead of 2D cell monocultures, a more realistic setup to study MNP uptake is the use of 3D models (e.g. spheroids, tumoroids, organoids) or a more complex but accurate approach like organ-on-a-chip setups where the *in vivo* environment, like the blood flow into capillaries, can be simulated into sophisticated microfluidic devices [207].

Furthermore, the ongoing advances in microscopy techniques offer several options to track the internalization and degradation of MNPs at a cellular level. For instance, fluorescence labeling of the plasma membrane can be used along with confocal microscopy to perform z-stacks to resolve the location of the MNPs in the cells. More sophisticated techniques include synchrotron radiation techniques with a spatial resolution that can go down to 30 nm, allowing both the localization and quantification of MNPs within cellular compartments and the local analysis of the structural and/or magnetic changes they undergo upon degradation. Among these techniques, cryo-soft x-ray tomography (cryo-SXT) allows 3D imaging of whole vitrified cells, thus closely preserving their native state and allowing distinguishing of the cell organelles and the MNPs [208]. X-ray fluorescence microscopy (XFM) on cellular sections provides simultaneously high-resolution, subcellular distribution and quantitative content of multiple elements. This can be combined with nanoscale-resolved absorption measurements (nano x-ray absorption near edge structure, nanoXANES [209]) allowing the identification of the new compounds appearing upon degradation of the MNPs. Additionally, the magnetic signal of individual or small clusters of MNPs can be tracked by scanning transmission x-ray microscopy (STXM) using x-ray magnetic circular dichroism (XMCD) as a contrast mechanism [210].

Lastly, regarding the advances in the control of the targeted delivery of MNPs, recent research is focusing on active MNPs designed as nanorobots. Notably, the utilization of biological entities, such as magnetotactic bacteria, to interact with tumors has become a growing area of interest, as they combine guidance, control, and therapeutic capabilities of MNPs with motility and environmental sensing [211].

### Concluding remarks

The physicochemical properties of MNPs affect their uptake by the cells as well as their degradation rate. New approaches to study MNP uptake alternative to pharmacological inhibitors are needed, and new 3D cell models will help us to better simulate what happens in the body. On the other hand, degradation of MNPs could decrease their therapeutic potential with important implications on the number of subsequent booster doses to be given to the patient. However, the release of Fe^2+^ during this process can also lead to ferroptosis, leaving open the possibility of using MNP for chemodynamic and ferroptosis therapies, which could be applied in combination with magnetic hyperthermia and photothermia. New techniques, such as spectromicroscopy synchrotron radiation techniques, are proposed to precisely localize and quantify MNP and identify their degradation products to better understand the degradation process and its implication in cancer treatment.

### Acknowledgments

This work was supported by the Spanish MCIN/AEI/10.13039/501100011033 under Project PID2020-115704RB-C31 and the Basque Government under project IT-1479-22. L.G. would like to acknowledge the financial support provided through a postdoctoral fellowship from the Basque Government (POS_2022_1_0017).

## Data Availability

No new data were created or analysed in this study.
